# Integrative analysis of omics summary data reveals putative mechanisms underlying complex traits

**DOI:** 10.1038/s41467-018-03371-0

**Published:** 2018-03-02

**Authors:** Yang Wu, Jian Zeng, Futao Zhang, Zhihong Zhu, Ting Qi, Zhili Zheng, Luke R. Lloyd-Jones, Riccardo E. Marioni, Nicholas G. Martin, Grant W. Montgomery, Ian J. Deary, Naomi R. Wray, Peter M. Visscher, Allan F. McRae, Jian Yang

**Affiliations:** 1 Institute for Molecular Bioscience, The University of Queensland, Brisbane, QLD 4072 Australia; 2 The Eye Hospital, School of Ophthalmology & Optometry, Wenzhou Medical University, Wenzhou, Zhejiang 325027 China; 3 Medical Genetics Section, Centre for Genomics and Experimental Medicine, Institute of Genetics and Molecular Medicine, University of Edinburgh, Edinburgh, EH4 2XU UK; 4 Centre for Cognitive Ageing and Cognitive Epidemiology, Department of Psychology, University of Edinburgh, 7 George Square, Edinburgh, EH8 9JZ UK; 5 Genetics and Computational Biology, QIMR Berghofer Medical Research Institute, Brisbane, QLD 4029 Australia; 6 Queensland Brain Institute, The University of Queensland, Brisbane, QLD 4072 Australia

## Abstract

The identification of genes and regulatory elements underlying the associations discovered by GWAS is essential to understanding the aetiology of complex traits (including diseases). Here, we demonstrate an analytical paradigm of prioritizing genes and regulatory elements at GWAS loci for follow-up functional studies. We perform an integrative analysis that uses summary-level SNP data from multi-omics studies to detect DNA methylation (DNAm) sites associated with gene expression and phenotype through shared genetic effects (i.e., pleiotropy). We identify pleiotropic associations between 7858 DNAm sites and 2733 genes. These DNAm sites are enriched in enhancers and promoters, and >40% of them are mapped to distal genes. Further pleiotropic association analyses, which link both the methylome and transcriptome to 12 complex traits, identify 149 DNAm sites and 66 genes, indicating a plausible mechanism whereby the effect of a genetic variant on phenotype is mediated by genetic regulation of transcription through DNAm.

## Abstract (web summary)

The identification of the causal gene at a GWAS locus remains to be a challenging task. Here, using the SMR & HEIDI method to integrate GWAS, eQTL and mQTL data, Wu et al. map DNA methylation sites to the transcriptome and thereby prioritize functionally relevant genes for 12 human complex traits.

## Introduction

Genome-wide association studies (GWAS) have identified thousands of genetic variants associated with complex traits (including diseases)^1,2^. Given the polygenic nature of most complex traits^3^, more variants are expected to be discovered in the foreseeable near future because of the rapid increase in sample size (due to large cohorts such as UK Biobank^4^ and consortia efforts). However, the mechanisms underlying the associations remain largely unaddressed^5^. This is because the mapping resolution of GWAS is limited by the complicated linkage disequilibrium (LD) structure of the genome (i.e., the top associated variant at a locus is often not the causal variant)^6,7^ and by the sampling variation in statistical tests due to finite sample sizes. Furthermore, genetic variants can affect phenotype through distal regulation of gene expression (i.e., the nearest gene to the GWAS top signal is often not the causal gene)^7–9^. High-throughput methods such as massively parallel reporter assay^10^, self-transcribing active regulatory region sequencing (STARR-seq)^11^, and CRISPR-Cas9 epigenome screens^12^ have been developed to identify the causal variants and functional elements regulating gene expression. However, it remains challenging to pinpoint the gene(s) responsible for the association signal at a GWAS locus^8,13,14^. Thus, analytical approaches that somehow mimic a functional study in silico are needed to prioritize plausible functional genes and/or regulatory elements at GWAS loci for further studies.

Given that the complex trait-associated variants are predominantly found in noncoding regions^7,15^, it is reasonable to assume that these variants affect phenotype through genetic regulation of transcriptional output. To test whether the effect of a genetic variant on a phenotype is mediated by transcription, we have developed powerful and flexible approaches, SMR (summary-data-based Mendelian randomization) and HEIDI (heterogeneity in dependent instruments) tests^9^, and have implemented them in an efficient software tool for genome-wide analysis (URLs). The SMR & HEIDI approach uses summary-level data from GWAS and expression quantitative trait locus (eQTL) studies to test if a transcript and phenotype are associated because of a shared causal variant (i.e., pleiotropy). Compared with most other methods for an integrative analysis of GWAS and eQTL data^16–18^, the SMR & HEIDI approach features the ability to distinguish a pleiotropic model (i.e., gene expression and phenotype are associated owing to a single shared genetic variant) from a linkage model (i.e., there are two or more distant genetic variants in LD affecting gene expression and phenotype independently)^9^. Moreover, like other summary-data-based methods^16,18^, the SMR & HEIDI approach allows the use of GWAS and eQTL data from two independent studies; thus, the statistical power can be boosted by using data from studies with very large sample sizes.

The analytical framework that integrates GWAS and eQTL data can be applied to incorporate other source of omics information, e.g., DNA methylation (DNAm) at CpG sites^19^, which is an important epigenetic mechanism for gene regulation^20^. Methylome-wide association studies (MWAS) have identified a number of DNAm sites associated with complex traits^21–23^. However, it is not clear whether these associations are causal or driven by confounding factors. More importantly, it is not obvious which genes are regulated by DNAm because the regulatory elements can be distant from the target genes^24^. Data from recent eQTL and methylation quantitative trait locus (mQTL) studies^25,26^ provide an opportunity to incorporate mQTL data into the SMR analysis to map DNAm to transcripts through a shared genetic factor and to further detect pleiotropic associations of DNAm and transcripts with phenotype. The chromatin activity data provided by the Roadmap Epigenomics Mapping Consortium (REMC)^27^ allows us to further annotate the DNAm sites that are associated with gene expression.

In this study, we report an integrative analysis of summary statistics from GWAS, eQTL and mQTL studies of the largest sample sizes to date. We mapped 7858 DNAm sites to 2733 putative target genes in *cis*-regions and then linked them to 14 complex traits.

## Results

### Method overview

**Fig. 1:**
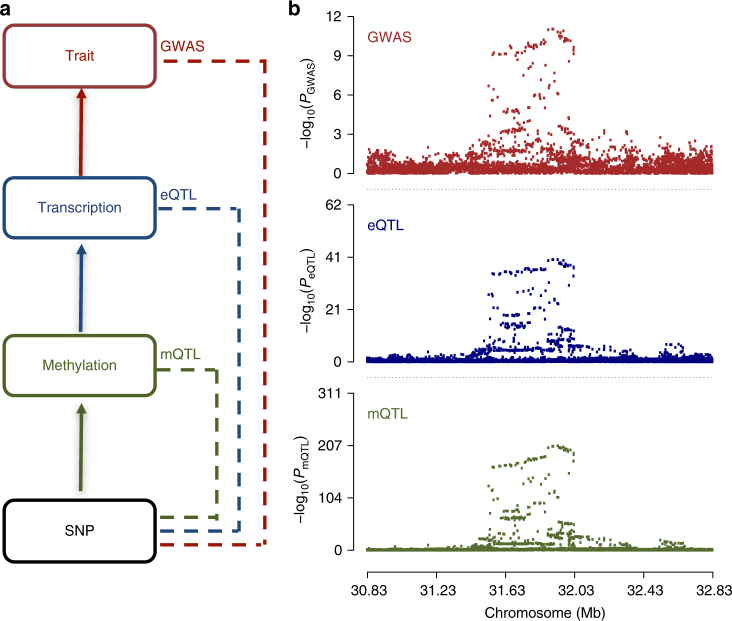
Schematic of our integrative analysis of multi-omics data. **a** A hypothetical model of a mediation mechanism tested in our analysis: an SNP exerts an effect on the trait by altering the DNAm level, which regulates the expression levels of a functional gene. **b** An example by simulation under our hypothetical model (Supplementary Note 1), in which the observed SNP-association signals are consistent at the shared GWAS locus across methylation, transcript and trait phenotype (see Fig. 7b for a real data example)

Our integrative analysis combines summary-level multi-omics data to prioritize gene targets and their regulatory elements. It consists of three steps, each of which relies on the SMR & HEIDI method to test for pleiotropic association^9^ (Fig. 1 and Supplementary Note 1). First, we map the methylome to the transcriptome in *cis*-regions by testing the associations of DNAm with their neighbouring genes (within 2 Mb of each DNAm probe) using the top associated mQTL as the instrumental variable (Methods). Next, we prioritize the trait-associated genes by testing the associations of transcripts with the phenotype using the top associated eQTL. Last, we prioritize the trait-associated DNAm sites by testing associations of DNAm sites with the phenotype using the top associated mQTL. If the association signals are significant in all three steps, then we predict with strong confidence that the identified DNAm sites and target genes are functionally relevant to the trait through the genetic regulation of gene expression at the DNAm sites. The SMR & HEIDI method assumes consistent LD between two samples. This assumption is generally satisfied by using data from samples of the same ancestry (Supplementary Fig. 1).

### Analytical mapping of methylome to transcriptome

To identify target genes for the DNAm sites, we applied the SMR & HEIDI approach to test for pleiotropic associations between DNAm and gene expression (denoted as M2T analysis) in large data sets from peripheral blood (Methods). The summary statistics of SNPs on gene expression were from an eQTL analysis of 38624 probes in the CAGE (Consortium for the Architecture of Gene Expression)^28^ data (*n* = 2765). The summary statistics of SNPs on DNAm were from a meta-analysis of mQTL data^26^ from two independent data sets: the Brisbane Systems Genetics Study (BSGS, *n* = 614)^29^ and the Lothian Birth Cohorts (LBC, *n* = 1366)^30^. After quality controls (Methods), we retained 9538 gene expression probes from the CAGE eQTL analysis and 73973 DNAm probes from the meta-mQTL analysis. In any of the specific analyses below, we only included SNPs available and with consistent alleles in the data sets used.

By testing each DNAm probe for associations with genes within a 2 Mb distance in either direction, we detected 21938 DNAm probes that showed significant SMR associations with 4804 gene expression probes (corresponding to 3991 unique genes) at *P*_SMR_ < 2.26 × 10^−8^ correcting for ~2.21 million tests. We further used the HEIDI approach to test against the null hypothesis that the association detected by the SMR test is due to pleiotropy, rejecting those with *P*_HEIDI_ < 0.01 (Supplementary Note 2). Finally, 10,588 associations between 7858 DNAm and 3239 gene expression probes (corresponding to 2733 genes) were not rejected by the HEIDI test. On average, 3.0 DNAm sites were associated with each gene, with a median value of 2.0 and a standard deviation of 3.1. For the DNAm sites associated with the same gene, they tended to be located proximally (average distance = 35.4 kb) and enriched in enhancers (see the enrichment analysis below), and their effects on the expression level of the gene tended to be in the same direction (87.2% pairs in the same direction). More than a half of the DNAm located in promoters (61.0%) or enhancers (62.2%) were negatively associated with the expression levels of the target genes, consistent with the hypothesis that most transcription factors are activators and the binding affinity of transcription factors on promoters and enhancers are affected by DNAm.

**Fig. 2:**
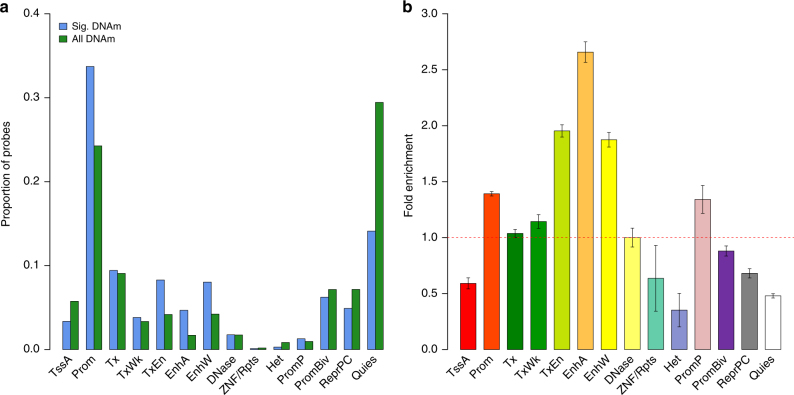
Enrichment analysis of transcript-associated DNAm probes identified by the SMR & HEIDI test for 14 main functional annotation categories. **a** Distribution of the transcript-associated DNAm probes ('Sig. DNAm', blue) across the 14 functional categories in comparison to that of all DNAm probes in the data ('All DNAm', green). **b** Fold enrichment: a comparison of the associated probes with the same probes sampled repeatedly at random with the variance of each probe matched. Error bar represents the standard error of an estimate obtained from 500 random samples. The 14 functional categories are: TssA active transcription start site, Prom upstream/downstream TSS promoter, Tx actively transcribed state, TxWk weak transcription, TxEn transcribed and regulatory Prom/Enh, EnhA active enhancer, EnhW weak enhancer, DNase primary DNase, ZNF/Rpts state associated with zinc-finger protein genes, Het constitutive heterochromatin, PromP Poised promoter, PromBiv bivalent regulatory states, ReprPC repressed Polycomb states, Quies a quiescent state

The SMR associations not rejected by the HEIDI test are consistent with a pleiotropic model whereby both DNAm and transcript are affected by a shared causal variant. We therefore hypothesized that the DNAm sites are located in regulatory elements that are functionally relevant to the associated genes. To test this hypothesis, we conducted an enrichment analysis (Methods) of the 7858 transcript-associated DNAm probes in 14 main functional annotation categories in blood samples from REMC (Methods)^27^. We found a significant enrichment of these DNAm probes in the promoter (fold-change = 1.39, *P* = 3.21 × 10^−88^) and enhancer (fold-change = 2.66, *P* = 2.20 × 10^−71^) regions and a significant underrepresentation in heterochromatin (fold-change = 0.35, *P* = 7.53 × 10^−6^), repressed (fold-change = 0.68, *P* = 2.79 × 10^−15^) and quiescent regions (fold-change = 0.48, *P* = 1.61 × 10^−157^), and transcription starting sites (fold-change = 0.59, *P* = 5.86 × 10^−17^) in comparison with the probes sampled at random with the variance of DNAm levels at each probe matched (Fig. 2). These results demonstrate the use of eQTL and mQTL summary data to genetically link a gene to its regulatory elements.

We also conducted a SMR & HEIDI analysis considering gene expression as the exposure and DNAm as the outcome (denoted as T2M analysis). The associations identified by M2T and T2M showed a substantial overlap (4182 associations in common) with 6406 and 3598 unique associations identified by M2T and T2M respectively (Supplementary Fig. 2), suggesting that M2T is more powerful than T2M. However, there were 248 associations identified by T2M, for which the instrument SNP explained significantly larger proportion of variance in gene expression than DNAm (Supplementary Data 1 and Supplementary Note 3), implying a potential mechanism that the genetic effect on DNAm is mediated through gene expression. The map between transcriptome and methylome identified in this study have been integrated in an online database (URLs), which is useful for interpreting the discoveries from MWAS^31^ and understanding the mechanism of genetic regulation of gene expression. The pleiotropic associations between DNAm and gene expression are driven by shared causal variants, and are therefore robust to environmental exposures or disease, unless the environmental exposures or diseases are dominated by a specific genotype, which is very unlikely for complex traits (Supplementary Fig. 3).

Genes in the closest physical proximity with the DNAm sites are often used as the target genes in MWAS. Given the DNAm–gene associations identified from the analysis above, we can estimate the proportion of DNAm sites mapped to the nearest genes. We found that only 36.3% of the DNAm sites were mapped to the nearest genes (*π*_nearest_) and 70.1% were mapped to distal (i.e., not the nearest) genes (*π*_distal_), with 6.4% mapped to both. There were a number of DNAm sites mapped to genes beyond 500 kb distance (Supplementary Fig. 4). For each of the distal DNAm–gene associations, we calculated the correlation in expression level between the nearest and distal genes. The mean correlation was only slightly above zero (mean *r* = 0.024, s.e.m. = 0.0020) and not significantly different from that of the same number of random gene pairs sampled from *cis*-regions with distances matched (mean *r* = 0.023, s.e.m. = 0.0033), not supporting the hypothesis that the distal associations are mediated through the nearest genes. Since not all the gene expression probes were included in the SMR analysis (we only included probes with *cis*-eQTL *P* < 5 × 10^−8^), *π*_nearest_ (*π*_distal_) could possibly be underestimated (overestimated) because some of the *cis*-eQTLs were not detected due to the lack of power. We then excluded 3368 DNAm probes for which the nearest genes were not included in the SMR analysis. The estimate of *π*_nearest_ increased to 63.6% and *π*_distal_ decreased to 47.6% (11.2% mapped to both). However, this stringent criterion would probably lead to an overestimation of *π*_nearest_ (underestimation of *π*_distal_) because some of the nearest genes do not have *cis*-eQTL even if the sample size is infinite. Nevertheless, our results indicate that at least 47.6% of the DNAm are involved in relatively distal regulation of the target genes, providing an important caveat that DNAm, such as those discovered in MWAS, do not always target the nearest genes. This result is supported by the observation from recent functional studies^8,32^ that the causal variants are largely located in regulatory sequences (e.g., enhancer regions) that are distal from the causal genes that they act on.

### Pinpointing functionally relevant DNAm and target genes

We applied the SMR & HEIDI method to perform pleiotropic associations of both the transcriptome and the methylome with 14 complex traits (including diseases). The summary statistics of SNP associations for the phenotypes were from the latest GWAS meta-analyses for complex traits^33,34,35^ and diseases^36–42^ (Methods and Supplementary Data 2). Using the *cis*-eQTL summary data of 9538 gene expression probes from the CAGE eQTL analysis described above, we performed the SMR test for associations between gene expression probes and each of the 14 traits, and identified 374 genes (tagged by 446 gene expression probes) at a genome-wide significance level (*P*_SMR_ < 6.97 × 10^−6^, i.e., 0.05/7177 tagged genes) for 13 traits (Supplementary Data 3). The HEIDI test rejected 152 of the associations detected by the SMR test (222 remaining) at a threshold 0.01 (Supplementary Data 3). Approximately, 65.6% of the 222 significant genes were not the nearest genes to the GWAS top associated SNPs, consistent with the result from a previous study^9^. We further developed a multi-SNP-based SMR method (SMR-multi) (Methods) and identified 564 genes at a genome-wide significance level, of which 235 were not rejected by the HEIDI test (Supplementary Data 4). The multi-SNP-based SMR test appeared to be more powerful than the single-SNP-based test (564 vs. 374). However, the number of SMR associations not rejected by the HEIDI test for the former was only mildly larger than that for the latter (235 vs. 222), suggesting SMR-multi picked up a large proportion of associations due to linkage. We therefore focused on the results from SMR and relegated those from SMR-multi in Supplementary Data 4. In the analysis with DNAm data, we used *cis*-mQTL summary data of 73973 DNAm probes from the meta-analysis described above and detected 1903 DNAm probes (Supplementary Data 5) associated with 14 complex traits at a genome-wide significance level (*P*_SMR_ < 6.81 × 10^−7^, i.e., 0.05/73448 where 73448 was the number of DNAm probes used in the analysis after matching the SNPs and alleles among the mQTL, GWAS and LD reference data), of which 893 DNAm probes were not rejected by the HEIDI test at *P*_HEIDI_ > 0.01.

**Fig. 3:**
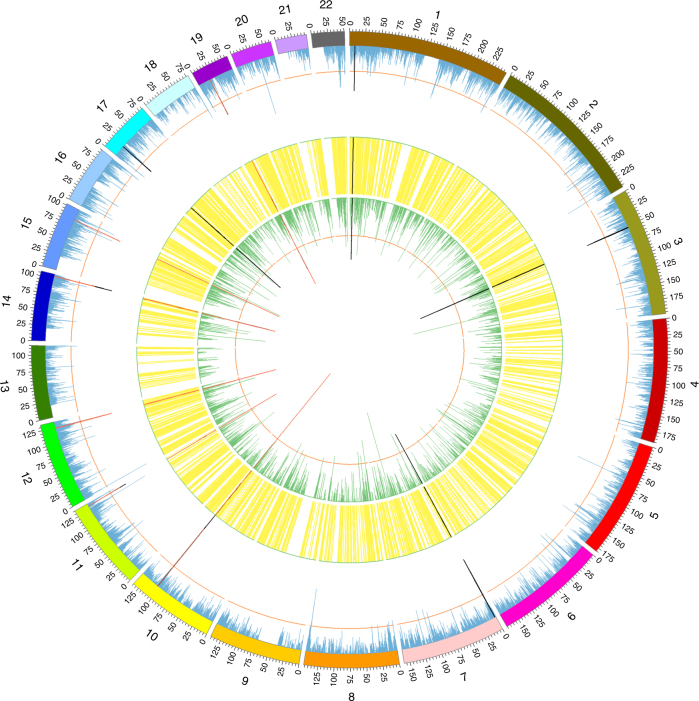
Pinpointing functional regions for a complex trait with consistent SMR association signals across multi-omics layers. Shown are −log_10_ (*P*-values) from the SMR tests for schizophrenia against the physical positions of DNAm or gene expression probes. The blue lines (outer ring) represent −log_10_ (*P-*values) from the SMR tests for associations between DNA methylation and trait, the green lines (inner ring) represent those for the associations between transcripts and trait and the yellow lines (middle ring) represent the significant associations between methylations and transcripts. The orange circles represent the significance thresholds of the SMR tests. The black lines are the significant SMR associations consistent across all three layers, and the red lines highlight the significant and consistent SMR associations that are not rejected by the HEIDI test. Due to the large scale of the plot, DNAm and genes that are distally associated (e.g. >500 kb and <2 Mb) appear to be closely located in the figure

Combining the results from pleiotropic associations between DNAm, transcripts and complex traits can potentially pinpoint functionally relevant genes and regulatory elements at GWAS loci, as demonstrated by the consistent association signals at the shared genomic regions across multiple omics layers (Fig. 3). When combining the results, we used a stringent criterion that both the DNAm site and transcript of each pair were associated with the trait at a genome-wide significance level with none of the associations rejected by the HEIDI test (see Supplementary Data 6 for details of the results from the SMR & HEIDI test). Of the 10588 DNAm–gene associations identified from the analysis above, 225 pairs (consisting of 149 DNAm and 66 genes) were significantly associated with 12 traits. Taking schizophrenia (SCZ) as an example, we found 10 genomic regions (tagged by 19 genes and 52 DNAm sites) with consistent significant association signals from DNAm sites and transcripts (Fig. 3). These results are in line with a model that the effects of genetic variants in these regions on SCZ susceptibility are mediated by the regulation of gene expression through DNAm (Fig. 1a). Among the 10 identified regions for SCZ, one on chromosome 12 (12q24.31) shows a pleiotropic effect on educational years (EY; Supplementary Fig. 5), consistent with a positive genetic correlation (*r*_g_) between SCZ and EY^43^. It should be noted that the gene–trait association analysis was not dependent on the DNAm–trait association analysis. There were 222 genes that showed pleiotropic associations with the traits, only 66 of which showed pleiotropic associations with DNAm.

Similar to the enrichment analysis of all transcript-associated DNAm sites shown above (Fig. 2), a subset of the 149 transcript- and trait-associated DNAm sites were also significantly enriched in the promoter and enhancer regions (Supplementary Fig. 6). Of the 66 identified genes, 22 genes are associated with diseases (Supplementary Data 7), i.e., Alzheimer’s disease (AD), rheumatoid arthritis (RA), SCZ, Crohn’s disease (CD), ulcerative colitis (UC) and coronary artery disease (CAD). To evaluate the potential value of the 22 putative disease susceptibility genes in drug discovery, we obtained all the drug target genes from a recent study^42^ based on two major drug databases, Drugbank^44^ and Therapeutic Targets Database^45^, which include drugs approved in clinical trials or experimental drugs. We found that five genes identified in our study (*MS4A2*, *FADS1*, *FADS2*, *LIPA*, *SULT1A1*) overlapped with the drug targets (Supplementary Data 8). For the drugs listed in Supplementary Data 8, those with estimated effects decreasing the disease risk might potentially be used as the candidates for drug repositioning, whereas those with estimated effects increasing the disease risk might need to be monitored for side effects in clinical trials or practice.

### Plausible mediation mechanism for susceptibility genes

**Fig. 4:**
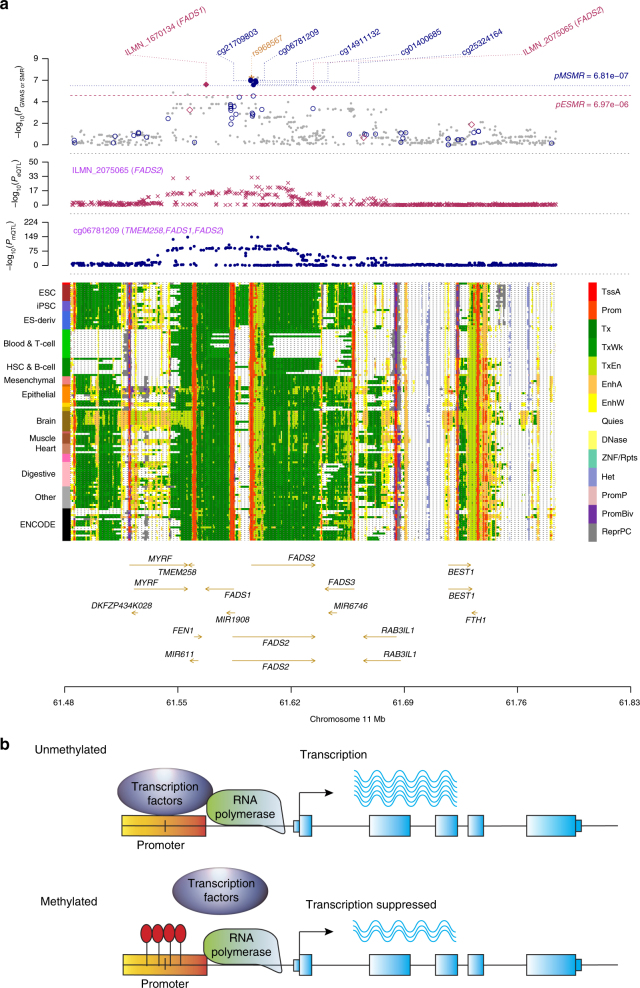
Prioritizing genes and regulatory elements at the *FADS1*/*FADS2* locus for rheumatoid arthritis (RA) with a plausible regulation mechanism. **a** Results of SNP and SMR associations across mQTL, eQTL and GWAS. The top plot shows −log_10_(*P*-values) of SNPs from the GWAS meta-analysis for RA^42^. The red diamonds and blue circles represent −log_10_(*P-*values) from SMR tests for associations of gene expression and DNAm probes with RA, respectively. The solid diamonds and circles are the probes not rejected by the HEIDI test. The yellow star indicates the previously reported causal variant rs968567. The second plot shows −log_10_(*P*-values) of the SNP association for gene expression probe ILMN_2075065 (tagging *FADS2*) from the CAGE eQTL study. The third plot shows −log_10_(*P*-values) of the SNP associations for DNAm probe cg06781209 from the mQTL study. The bottom plot shows 14 chromatin state annotations (indicated by colours) of 127 samples from REMC for different primary cells and tissue types (rows). **b** A hypothetical regulation mechanism. When the DNAm site in the promoter is unmethylated, the transcription factor *SREBF2* (activator) binds to the promoter and enhances the transcription of the *FADS2* gene. When the DNAm site is methylated (by the effect of the genetic variant rs968567 at the promoter), the binding of *SREBF2* is disrupted and therefore the transcription of *FADS2* is suppressed

The discovery of putative functional genes and their regulatory elements in our analysis provides opportunities to infer the mechanism of genetic regulation at a GWAS locus. A notable example is the DNAm that reside in the promoter region within *FADS2* for RA (Fig. 4a), where the SNP-association signals are significant and consistent across data from GWAS, eQTL and mQTL studies, implying a plausible biological pathway. We detected two gene expression probes (ILMN_2075065 and ILMN_1670134) tagging *FADS1* and *FADS2* that are significantly associated with RA. The SNP-association signals for gene expression coincide with those for the five nearby DNAm probes (cg06781209, cg21709803, cg01400685, cg25324164 and cg14911132) that are mapped to the two genes, suggesting that the two genes are co-expressed (estimated correlation of 0.56 in the GTEx blood samples) and potentially regulated by these five DNAm sites. The co-regulation hypothesis is supported by the evidence that both *FADS1* and *FADS2* are involved in the metabolism of omega-6 and omega-3 fatty acids^46^ (Supplementary Data 7). Indeed, the five DNAm sites that are significantly associated with *FADS1* and *FADS2* are in the promoter and nearby enhancer regions of the two genes according to the chromatin state annotations from the REMC^28^ reference samples. Furthermore, rs968567 is the top associated SNP in both the eQTL analysis of the two genes and the mQTL analysis of the five DNAm (Supplementary Data 6). This variant is only 567 bp away from one of the five DNAm sites and has been identified in a recent study^47^ as the binding site of transcription factor *SREBF2*. The negative estimates of methylation effects on gene expression from SMR (*b*_SMR_ = −0.812, Supplementary Data 6) indicate a repressing role of DNAm on target gene expression. With all the evidence above, we hypothesize a mechanism in which the genetic variant (rs968567) at the promoter of *FADS2* gene alters the DNAm, which disrupts the binding of transcription factor (*SREBF2*), down-regulating the expression of the *FADS2* gene and therefore decreasing the risk of RA^47^ (Fig. 4b).

**Fig. 5:**
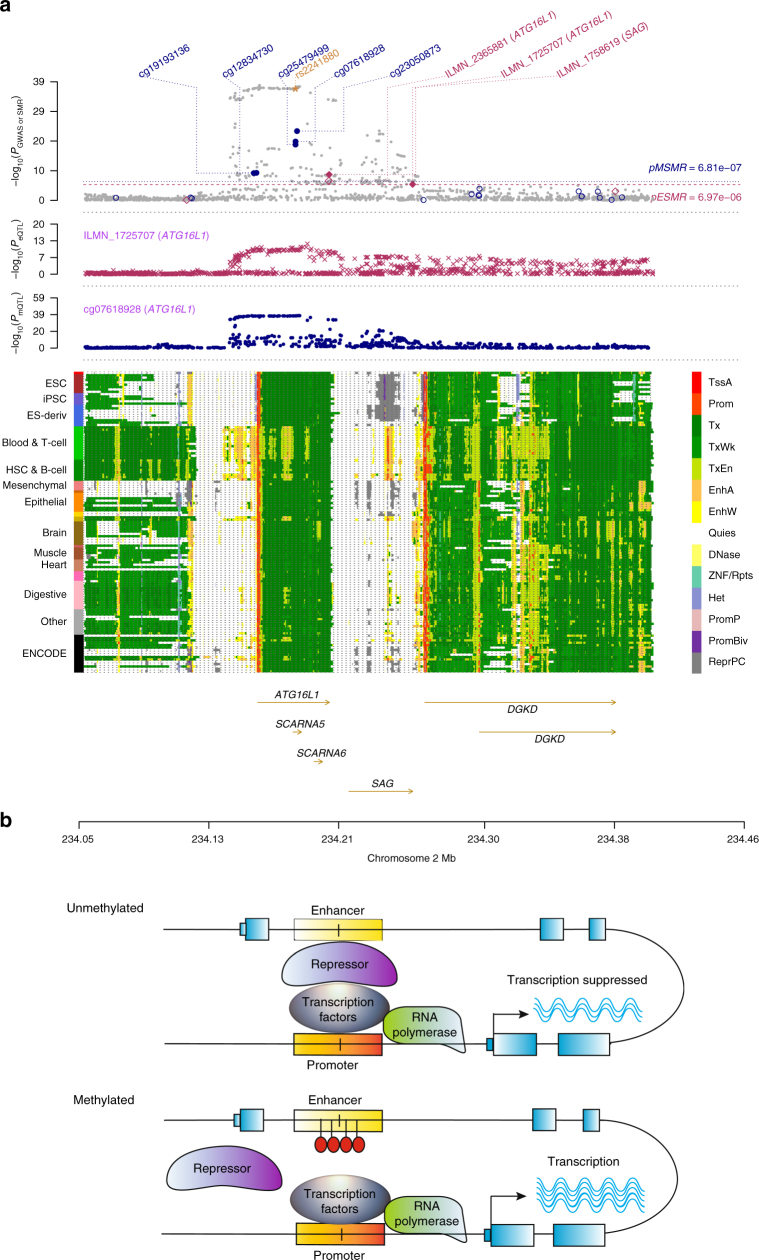
Prioritizing genes and regulatory elements at the *ATG16L1* locus for Crohn’s disease (CD) with a plausible regulation mechanism. **a** Results of SNP and SMR associations across mQTL, eQTL and GWAS. The top plot shows −log_10_(*P-*values) of SNP from the GWAS meta-analysis for CD^40^. The red diamonds and blue circles represent –log_10_(*P-*values) from the SMR tests for associations of gene expression and DNAm probes with CD, respectively. The solid diamonds and circles represent the probes not rejected by the HEIDI test. The yellow star indicates the previously reported causal variant rs2241880. The second plot shows −log_10_(*P*-values) of the SNP associations for gene expression probe ILMN_1725707 (tagging *ATG16L1*). The third plot shows −log_10_(*P-*values) of the SNP associations for DNAm probe cg07618928. The bottom plot shows 14 chromatin state annotations (indicated by colours) of 127 samples from REMC for different primary cells and tissue types (rows). **b** A hypothetical regulation mechanism. When the DNAm site in the enhancer is unmethylated, repressors can bind to the enhancer, decrease the activity of the promoter, and thus suppress the transcription of the *ATG16L1* gene. When the DNAm site is methylated (by the effect of the genetic variant rs2241880 in the enhancer), the binding of repressors is disrupted, which prevents the transcription of *ATG16L1* from suppression

The *ATG16L1* gene for CD is another interesting example of inferring a plausible mechanism of genetic mediation from the SMR analysis of omics data. As shown in Fig. 5a, there are five DNAm sites that are significantly associated with *ATG16L1* and CD, among which two are in the promoter region and three are in an enhancer region within the open reading frame (ORF) of *ATG16L1*. This enhancer is highly tissue-specific and is only present in the blood, thymus, digestive system and a few obscure samples in REMC; most of these tissues are relevant to CD. An intronic variant rs2241880 in *ATG16L1*, which has been confirmed to be the causal variant for CD and has been validated at the protein level^48^, is only 56 bp away from one of the associated DNAm sites in the tissue-specific enhancer region (Supplementary Data 6). All the results point strongly towards a mechanism of genetic regulation of gene expression initiated by DNAm in the enhancer and mediated through enhancer–promoter interactions at the *ATG16L1* locus for CD (Fig. 5b). Furthermore, the identified DNAm in the enhancer and promoter regions all have positive effects on gene expression (Supplementary Data 6), suggesting that the transcription factors that bind to these regions are repressors (Fig. 5b).

One further example is the regulatory mechanism of a gene (i.e., *LIPA*) associated with CAD. A previous study has shown that an exonic variant rs1051338 in *LIPA* decreases lysosomal acid lipase levels and activity in lysosomes^49^. Interestingly, we found that the top associated DNAm is located in an enhancer region and that the top mQTL is only 110 bp away from rs1051338 (Supplementary Fig. 7).

We have shown, as a proof-of-principle, the examples above where the functional genes and the likely mechanisms are known or strongly supported by previous studies. However, these examples are rare. We have identified 66 genes and 149 DNAm that show pleiotropic associations with 12 traits. These results demonstrate the power of re-analysing and integrating different levels of data from published studies of large sample size to make novel discoveries and to shed light on putative causal genes and regulatory elements that can be prioritized in functional studies.

### Pleiotropic effects of DNAm, transcripts and traits

Previous studies have used GWAS data to characterize genetic overlap between human complex traits and common diseases^43,50^. In our integrative analysis, we detected several DNAm sites and/or transcripts that show pleiotropic effects on multiple traits (Supplementary Data 9), especially traits that are known to be genetically correlated. Shown in Supplementary Fig. 8 is an example in which the *ARL6IP4* locus shows pleiotropic effects on EY and SCZ (estimated *r*_g_ from genome-wide SNPs^51^ of 0.10, *P* = 8.47 × 10^−3^), where the SNP-association signals from the mQTL and eQTL studies are highly consistent with those from GWAS for EY and SCZ. We have examples where the effect sizes of a transcript on two traits are in the same direction. For example, height shares genes (*SLC22A4* and *SLC22A5*) with CD, and the effects of transcripts on the two traits are in the same direction, which is accordant with the positive genetic correlation (*r*_g_ = 0.06, *P* = 0.12) between two traits although the estimate of *r*_g_ is very low and not significant. We also observed pleiotropic effects in the opposite direction for two negatively correlated traits, e.g., height and low-density lipoprotein (LDL) (*r*_*g*_ = −0.09, *P* = 8.02 × 10^−3^), at three genes (*PLEC*, *GRINA* and *PARP10*) (Supplementary Fig. 9). Among all 14 traits, height shares the largest number of genes with other traits, which is likely due to the relatively larger GWAS sample size and the polygenic architecture. Not surprisingly, the pairs of traits that share more than three pleiotropic genes are those that have been shown to be genetically correlated^43^, e.g., height and LDL (*r*_*g*_ = −0.09, *P* = 8.02 × 10^−3^), CD and UC (*r*_*g*_ = 0.54, *P* = 1.69 × 10^−13^), and EY and height (*r*_*g*_ = 0.13, *P* = 3.82 × 10^−6^). Nevertheless, this concordance depends on the sample sizes of the GWAS data.

### Robustness and tissue specificity

Having identified many pleiotropic associations between DNAm, gene expression, and complex traits, we then sought to investigate how robust the results are across tissues and data sets. We used the same data-filtering criteria (Methods) for a replication analysis using eQTL summary data from whole-blood samples in Westra et al.^26^ and for a tissue-specific analysis using eQTL summary data from brain samples in the Common Mind Consortium (CMC)^52^, Genotype-Tissue Expression (GTEx)^53^ and the Brain eQTL Almanac (Braineac)^54^. We performed the SMR analysis using eQTL summary data of 5967 gene expression probes after quality controls from the Westra et al. study^26^, which has a larger sample size (*n* = 5311) than CAGE. We used CAGE for discovery because of its denser SNP coverage (SNPs in Westra were imputed to HapMap2 with a maximum of ~2.5 M, and only SNPs with *P*_eQTL_ < 1 × 10^−5^ are available). For the significant DNAm–transcript associations identified using the CAGE data, the SMR estimates of the effect sizes of DNAm on transcripts were highly consistent with those estimated in the Westra data (Supplementary Fig. 10) with an estimated correlation of 0.98. A total of 24360 (63%) significant DNAm–transcript associations detected using the CAGE data were significant at *P*_*SMR*_ < 2.90 × 10^−7^ in the analysis with the Westra eQTL data. Although there is a sample overlap between CAGE and Westra, this analysis demonstrates the robustness of the results across data sets generated from different studies.

**Fig. 6:**
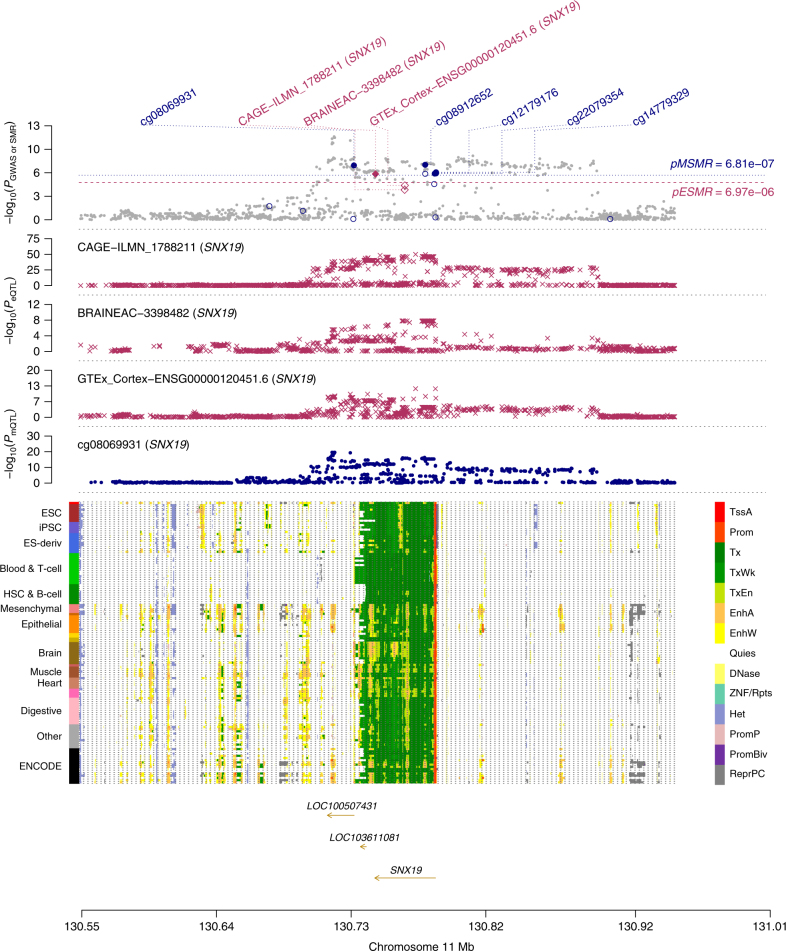
Replication analysis in the *SNX19* locus for schizophrenia (SCZ). The top plot shows −log_10_(*P-*values) of the SNPs from the GWAS meta-analysis for SCZ^36^. The red diamonds and blue circles represent −log_10_(*P-*values) from the SMR tests for associations of gene expression and DNAm probes with SCZ, respectively. The solid diamonds and circles represent the probes not rejected by the HEIDI test. Plot #2, #3 and #4 (red crosses) show −log_10_(*P-*values) of the eQTL for gene *SNX19* from three independent data sets. The plot #5 (blue dots) shows −log_10_(*P*-values) of the mQTL for DNAm probe cg08069931. The bottom plot shows 14 chromatin state annotations (indicated by colours) of 127 samples from REMC for different primary cells and tissue types (rows)

In addition, we used data from the blood samples as the discovery set because of the large sample sizes, which maximizes the power of discovery. To test whether eQTL and mQTL data from the blood sample are good representatives of those from the most relevant tissue, we performed the SMR analysis for SCZ and EY using three eQTL data sets from the brain (i.e., CMC, GTEx and Braineac). In total, there are nine genes (56%) replicated for SCZ and seven genes (77%) replicated for EY in at least one of the three brain eQTL data sets (Supplementary Data 10). These replication rates are surprisingly high given the relatively small sample sizes of the brain eQTL studies (*n* = up to 467). Genes *SNX19*, *NT5C2* and *MAPK3* for SCZ and the genes *ERCC8* and *C18orf8* for EY (Supplementary Fig. 11) were replicated in at least two data sets. *MAPK3* was highlighted in a recent study^55^ as a functional gene whose expression is up-regulated by a variant by disrupting chromatin activity, which is associated with a decrease in SCZ risk. Consistent with our previous result^9^, we again detected the association of *SNX19* with SCZ in the GTEx data. Figure 6 shows that the eQTL association signals for *SNX19* from the three data sets of two different tissues are consistent with the GWAS association signals for SCZ, despite that the gene expression levels were measured by different technologies (gene expression microarray for Braineac and RNA sequencing for GTEx). We further identified five DNAm probes that were associated with both *SNX19* and SCZ at promoter and enhancer regions close to *SNX19* (Fig. 6). Together with that *SNX19* is the only annotated gene underlying this GWAS locus, the results suggest a likely mechanism that a genetic variant (of course, may not be the GWAS top SNP) alters the methylation level of a DNA element that regulates the expression level of *SNX19*, resulting in a small difference in susceptibility to SCZ.

## Discussion

**Fig. 7:**
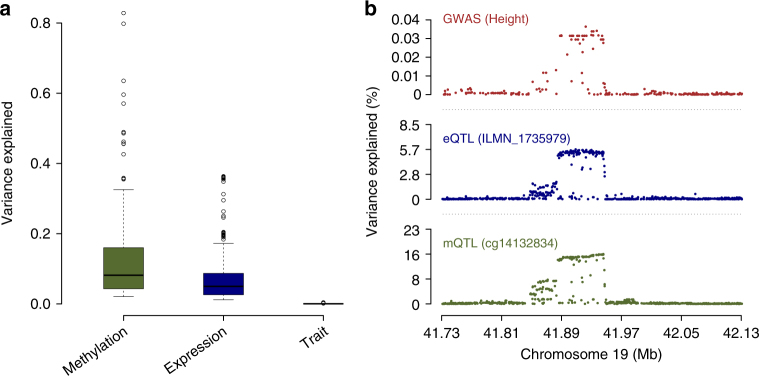
The attenuation of effect sizes of genetic variants on methylation and gene expression towards complex traits. **a** Distribution of the variance explained in methylation, gene expression and trait phenotypes by the top mQTLs for the 149 DNAm that are significantly associated with both 66 transcripts and 12 traits. Of 225 DNAm–transcript pairs, there are 160 pairs for which the mQTL effect is higher than the eQTL effect (both in SD units). **b** An example of a genomic locus for height, where the SNP-association signals are consistent across mQTL, eQTL and GWAS, indicating a single shared underlying causal variant, but the variance explained decreases dramatically across these studies

We introduced an integrative analysis based on the SMR & HEIDI method to map DNAm sites to putative target genes and further map both to a complex trait. We tested a model at each GWAS locus to evaluate whether an SNP exerts an effect on the trait by altering the DNAm level, which regulates the expression level of a functional gene (Fig. 1a). This hypothetical model is supported by many examples of consistent SMR associations across DNAm, transcript and complex traits in our analysis. It is also consistent with our observation that the variance explained by the top associated mQTL decreases dramatically from that for DNAm to gene expression and to phenotype (Fig. 7).

In this study, we identified 7858 DNAm probes associated with 2733 genes, of which 149 DNAm loci and the corresponding 66 putative target genes are associated with 12 complex traits and diseases, through pleiotropy. As expected, the DNAm that show pleiotropic associations with genes are enriched in the enhancer and promoter regions. Notably, 48–70% of the DNAm sites do not show pleiotropic association with their closest genes, indicating that a large proportion of DNAm sites are in distal functional regions (e.g., enhancer) and regulate the target genes probably through interactions of looping chromatins^56^. Furthermore, DNAm in enhancer and promoter regions is presumed to be involved in the transcription factor binding process^57^, which implicates the likely role of the causal variants as binding sites. Given the direction of DNAm effect on gene expression, we can further infer the role of the transcription factor as an activator or repressor. In this study, we found that a substantial number of DNAm sites (46%) have positive effects on their target genes, implying that the transcription factors that bind to the unmethylated DNA at these sites are likely to be repressors. We replicated several putative causal genes for complex traits reported in previous studies (e.g., *ATG16L1* for CD and *LIPA* for CAD)^48,49^. However, the causal genes for body mass index (BMI) (i.e., *IRX3* and *IRX5*) were not detected in our study, likely because the effects of the *FTO* SNP on *IRX3* and *IRX5* are specific in primary preadipocytes (a minority group of adipose cells)^8^ whereas the eQTL data used in our study were from peripheral blood. In summary, based on the methylome-transcriptome map, the incorporation of DNAm–trait and transcript–trait association information contributes to an understanding of the mediation mechanism of genetic variants on complex traits and diseases.

Under the analytical paradigm introduced in this study, it is straightforward to incorporate other types of molecular traits from specific cells. For instance, RNA splicing can be an important source in the differentiation of gene expression. One recent study showed that splicing QTLs (sQTLs) contribute remarkably to complex trait variation, roughly as much as that from eQTLs^58^. An integrative analysis that combines sQTL data is expected to shed light on the underlying mechanism of differential expression. Other types of epigenetic data, such as chromatin phenotypes^59^, can be further used to detect epigenetic interactions and decipher gene regulations in three dimensions.

Although a tissue-specific mechanism has been proposed to explain the differences in gene expression levels across different primary cells and tissues, we showed that a large proportion of the detected genes for SCZ and EY using peripheral blood samples is replicated in brain tissues. Using the data in a single tissue with a large sample size allows us to gain power in the discovery of novel trait-associated genes and regulatory elements. On the other hand, the power of the SMR test largely depends on the sample size of GWAS because of the small effect sizes of SNPs on complex traits (Fig. 7). For example, no gene expression probe and only eight methylation probes were significantly associated with T2D in the SMR test, likely a consequence of the limited sample size of GWAS for T2D. In addition, only a subset of probes were used in the SMR analysis after the filtering criterion that at least one SNP is significantly associated with the transcript (*P*_eQTL_ <  5 × 10^−8^). A proportion of transcripts may be further missed due to the elimination of expression probes with inconsistent gene mapping when combining eQTL data across multiple studies (e.g., CAGE), resulting in a potential loss of power. Given that future eQTL and mQTL studies will have larger sample sizes, we expect to detect more genes and DNAm sites that show pleiotropic associations with complex traits and diseases through our integrative analysis.

There is a limitation of this study. That is, we performed the SMR & HEIDI analysis to detect DNAm–gene, DNAm–trait and gene–trait associations separately, and focused on the association signals that were consistent across the three analyses at a locus. This strategy, however, is not optimal and potentially loses power because of thresholding the results by *P*-values in multiple steps. Further development of the method that integrates GWAS data and all the omics data in a single test is a priority in the future (Supplementary Note 4). Despite this limitation, our study provides a statistically elegant analytical paradigm that integrates genomic, transcriptomic and epigenomic information to understand the regulatory mechanism of polygenic effects for complex traits. The methylome-transcriptome pleiotropic map constructed in this study will be helpful for researchers to query the putative target gene(s) of a given DNAm site, and the database (URLs) can be expanded in the future with more data. The analyses we perform here can be applied to any other source of omics data and any other phenotype or disease, even in a different species, using the tools that we have made available (URLs). The putative functional genes and regulatory elements identified in this study provide important leads for designing studies in the future to understand the mechanism of genetic regulation of genes affecting common complex diseases.

## Methods

### SMR and HEIDI test for pleiotropic association

The SMR test^9^ was developed to test the association of an exposure (e.g., transcript) with an outcome (e.g., trait) using a genetic variant as the instrumental variable to remove non-genetic confounding. Let *x* be an exposure variable, *y* be an outcome variable, and *z* be an instrumental variable. What these variables refer to varies in different steps of our analysis. In the step of testing for a DNAm–gene association, *x*, *y* and *z* refer to DNAm, transcript and the top-associated mQTL, respectively. In the steps of testing for a DNAm/transcript–trait association, *x*, *y* and *z* refer to DNAm/transcript, trait and the top-associated mQTL/eQTL, respectively. The Mendelian Randomization (MR) estimate of the effect of exposure on outcome ($\hat b_{xy}$) is the ratio of the estimated effect of instrument on exposure ($\hat b_{zx}$) and that on outcome ($\hat b_{zy}$)^9,60,61^:

$$\hat b_{xy} = \hat b_{zy}/\hat b_{zx},$$

where $\hat b_{zx}$ and $\hat b_{zy}$ are available from mQTL, eQTL or GWAS summary data. One of the basic assumptions for MR is that the instrument should be strongly associated with exposure. We therefore only select the top associated eQTL/mQTL at *P* < 5 × 10^−8^ as an instrument for an SMR analysis. The standard error (SE) of $\hat b_{xy}$ can be computed from the SEs of $\hat b_{zx}$ and $\hat b_{zy}$ using the Delta method^9,61^. The significance of $\hat b_{xy}$ can therefore be assessed by the Wald test, i.e., $\left( {\frac{{\hat b_{xy}}}{{{\mathrm{SE}}}}} \right)^2\sim \chi _1^2$.

A significant association detected by the SMR test above can result from either a pleiotropic model (i.e., the exposure and the outcome are associated owing to a single shared genetic variant) or a linkage model (i.e., there are two or more genetic variants in LD affecting the exposure and outcome independently). To distinguish pleiotropy from linkage, the HEIDI (heterogeneity in dependent instruments) test was developed to test against the null hypothesis that there is a single causal variant underlying the association (pleiotropy)^9^. In brief, we use multiple SNPs (e.g., the top 20 associated mQTLs/eQTLs after pruning SNPs for either too strong or too weak LD^9^) in a *cis* region to detect whether the association patterns across the region are homogeneous or not (a homogenous pattern indicates a single shared causal variant). That is, we assess the difference between $\hat b_{xy}$ estimated at the top associated instrument $\left( {\hat b_{xy(0)}} \right)$ and $\hat b_{xy}$ estimated at a less significant instrument $\left( {\hat b_{xy\left( i \right)}} \right)$:

$$\hat d_i = \hat b_{xy\left( i \right)} - \hat b_{xy\left( 0 \right)}.$$

For multiple SNPs, $\widehat {\bf{d}}\sim \mathbf{MVN}\left( {{\bf{d}}{\mathrm{,}}{\bf{V}}}\right)$, where $\widehat {\bf{d}} = \left\{ {\hat d_1, \cdots ,\hat d_m} \right\}$ and **V** is the covariance matrix because most SNPs are likely in LD^9^. In this study, we estimated LD from the Health and Retirement Study (HRS)^62^ with SNP data imputed to the 1000 Genomes Project (1KGP)^63^. Under the null hypothesis (pleiotropic model), **d** = **0**. We use an approximate multivariate approach to test whether **d** is significantly deviated from **0** (equivalent to a test for evidence of heterogeneity in $\hat b_{xy}$ estimated at multiple instruments that are likely in LD)^9,64^. We reject the SMR associations that show significant heterogeneity as detected by the HEIDI test.

### Multi-SNP-based SMR test

We can extend the SMR method by including multiple SNPs at a *cis*-mQTL/eQTL locus in the SMR test. We call this method as SMR-multi and have implemented in the SMR software (URLs). First, we select all the SNPs with *P < *5 × 10^−8^ in the *cis* region (e.g. within 500 kb of the probe) and remove SNPs in very high LD with the top associated SNP (e.g. LD *r*^2^ > 0.9). We then estimate *b*_*xy*(*i*)_ at each of the SNPs and combine the *b*_*xy*_ estimates of all the SNPs in a single test using an approximate set-based test developed previously^64^ accounting for LD among SNPs. In brief, let **z** = {*z*_*i*_} be a vector of *z*-statistics for all the instruments at a locus, where $z_i \approx b_{xy\left( i \right)}/{\mathrm{SE}}$. Under the null hypothesis, **z** follows a multivariate normal distribution, i.e. **z** ~ MVN(**0**, **R**), where **R** is the LD correlation matrix for the SNPs at a locus. For the significance test, we use the test-statistic $T = \mathop {\sum}\nolimits_i {z_i^2}$, which does not have an explicit cumulative density function but can be approximated by the Saddlepoint method^64,65^.

### Data used for the integrative analysis and quality controls

The eQTL summary-level statistics were from the CAGE^29^ data, which consists of a total of seven distinct cohorts with gene expression levels measured in peripheral blood. This unified dataset comprises 2765 individuals (predominantly Europeans), 38624 normalized gene expression probes and ~8 million SNPs. The eQTL effects were in standard deviation (SD) units of transcription levels. For replication, we used eQTL summary-level data from the Westra et al.^26^ study, which is a meta-analysis of 5311 blood samples based on SNP data imputed to HapMap2 (ref.^66^). Gene expression in these two data sets was mainly measured based on Illumina HumanHT-12 v3.0 chip, and the annotations were from Illumina based on hg18 (see URLs). We used the function illuminaHumanv4fullReannotation in Bioconductor^67^ to update the annotation of the gene expression probes based on hg19. Next, we selected the probes with good tagging of gene expression (i.e., a probe annotation quality score of at least 'good'^68^) and at least one *cis*-eQTL passing the significant threshold (*P*_eQTL_ < 5 × 10^−8^). The eQTL effect sizes were not available in Westra data, but they were estimated from *z*-statistics using the method described in Zhu et al.^9^. Probes at the major histocompatibility complex (MHC) region were excluded because of the complexity of this region. We also removed probes with SNPs in the hybridization sequences. Finally, we retained 9538 and 5967 gene expression probes from CAGE and Westra, respectively, for analysis.

The mQTL data were from the Brisbane Systems Genetics Study^30^ (*n* = 614) and Lothian Birth Cohorts of 1921 and 1936^31^ (*n* = 1366). All the individuals are of European descent. The methylation states of all the samples were measured based on Illumina HumanMethylation450 chips, consisting of 485512 DNAm probes. For these probes, an enhanced annotation from Price et al.^69^ (see URLs) was used to annotate the closest genes of DNA methylation probes. The summary-level statistics of the two independent cohorts were obtained from a recent mQTL study^27^, where the mQTL effects were in SD units of DNAm levels. There were ~8 million genetic variants after quality control and 55000 mQTL were cross-replicated in the two data sets at a very stringent significance level. We performed a meta-analysis of the two cohorts and identified 94338 methylation probes with at least a *cis*-mQTL at *P*_mQTL_ < 5 × 10^−8^. We excluded methylation probes in MHC regions and probes with SNPs in the hybridization sequences, resulting in 73973 probes retained for analysis.

We included in the analysis 14 complex traits (including disease). They are height, BMI, waist–hip ratio adjusted by BMI (WHRadjBMI), high-density lipoprotein, LDL, thyroglobulin, EY, RA, SCZ, CAD, type 2 diabetes (T2D), CD, UC and AD. The GWAS summary data were from the latest GWAS meta-analyses (predominantly in Europeans) at the time when the analyses were performed, where the sample sizes are up to 339224 (Supplementary Data 2). The number of SNPs varies from 2.5 to 9.4 million across traits. The SNP effects on quantitative traits were in SD units, whereas those on disease traits (e.g., case-control design) were expressed as log odds-ratios. For those traits (i.e., RA and SCZ) for which the SNP allele frequencies are not available, we estimated the allele frequencies using the 1KGP-imputed HRS data. We further excluded variants with a minor allele frequency <0.01.

### Chromatin state annotation

The epigenomic annotations as shown in Figs 4–6 are from the Roadmap Epigenomics Mapping Consortium (REMC), which is publicly available for download at http://compbio.mit.edu/roadmap/. We use these annotations to indicate the regulatory elements and cell and tissue types where the functional DNAm sites and causal variants might act. The chromatin state data of 127 epigenomes were profiled by the Roadmap Epigenomics Project^28^ and ENCODE Project^70^ in a number of primary cells and tissue types (URLs). The 25 chromatin states were predicted by ChromHMM^71^ based on the imputed data of 12 histone-modification marks. The 14 main functional categories such as those shown in Fig. 4 and the enrichment analysis below were derived from the 25 chromatin states by combining functionally relevant annotations to a single functional category (Supplementary Table 1).

### Enrichment test of functional categories

We used the chromatin state annotation of 23 blood cell types from 127 epigenomes described above to test whether the transcript-associated DNAm are enriched in any functional region(s). We quantified the proportion of overlap between the transcript-associated DNAm probes and 14 main functional categories (see Fig. 2). To calibrate the distribution under the null of no enrichment, we repeated the analysis with the same number of DNAm probes randomly sampled from all probes, matching their variance in DNAm levels with each of the transcript-associated DNAm probes. This procedure was performed over 500 times, and the fold enrichment was calculated by a comparison of the observed value with the mean from 500 null replicates. To assess the significance of the enrichment test, we estimated the standard error of the fold enrichment from 100 null replicates.

### Tissue specificity analysis from brain samples

To detect the tissue specificity for brain-related traits or disease, we performed the SMR analysis using eQTL summary data from the Common Mind Consortium (CMC) and Genotype-Tissue Expression (GTEx). Gene expression levels of these two data sets were quantified by RNA sequencing, and the annotation was from GENCODE Version 19 (ref.^72^). The GTEx summary data are available at dbGaP (URLs), and the sample size of the brain tissue is up to 125. It consists of up to 24762 transcripts in 10 brain regions and up to ~6.5 million 1KGP-imputed variants. We carried out the SMR analysis for each brain region and counted the result as a successful replication if a significant SMR result from the analysis with the CAGE data was detected in any of the 10 brain regions after correcting for multiple tests. We further validated the results using the CMC data, which were generated from 467 brain samples. Gene expression levels were measured across multiple regions of the whole brain. The eQTL summary data were from the analysis of 16423 transcripts and ~2 million 1KPG-imputed variants. We also performed the SMR analysis with Braineac eQTL summary data, which are available in the public domain (see URLs) and comprise 26493 gene expression probes in ten brain regions and ~6 million genetic variants on up to 134 individuals.

### URLs

For M2Tdb, see http://cnsgenomics.com/shiny/M2Tdb/. For SMR, see http://cnsgenomics.com/software/smr. For GTEx, see http://www.gtexportal.org/home/. For CMC, see https://www.synapse.org/CMC. For Braineac, see http://www.braineac.org/. For the Annotation file for the Illumina HumanHT-12 v3.0 Gene Expression BeadChip, see https://support.illumina.com/downloads/humanht-12_ v3_product_files.html. For the

Annotation file for the Illumina HumanMethylation450 BeadChip, see. https://www.ncbi.nlm.nih.gov/geo/query/acc.cgi?acc=GPL16304.

### Data availability

The summary-level eQTL data from the CAGE and mQTL data from the meta-analyses of LBC and BSGS are available at http://cnsgenomics.com/software/smr/#Download. All the other data sets used in this study are from the public domain. The software tools are available at the URLs above.

## Electronic supplementary material

Supplementary Information

Description of Additional Supplementary Files

Supplementary Data 1

Supplementary Data 2

Supplementary Data 3

Supplementary Data 4

Supplementary Data 5

Supplementary Data 6

Supplementary Data 7

Supplementary Data 8

Supplementary Data 9

Supplementary Data 10
