# Supplementary material for: Integrative analysis of omics summary data reveals putative mechanisms underlying complex traits
Source: Nat Commun. 2018 Mar 2;9:918. doi: 10.1038/s41467-018-03371-0 (PMC5834629; doi:10.1038/s41467-018-03371-0)
Supplement: Supplementary file 1 — Supplementary Information [file 41467_2018_3371_MOESM1_ESM.pdf]

Supplementary Note 1: Simulation studies

We conducted the following two sets of simulations based on whole genome sequencing (WGS) data from the UK10K study[1]. One is used to demonstrate our hypothetical model of causality, and the other is to determine the minimum number of SNPs that maximizes the power of the HEIDI test. In both sets of simulations, we only included unrelated individuals (*n* = 3,642) and ~8.3 million SNPs with (MAF) > 0.01 and Hardy-Weinberg Equilibrium (HWE) *P* value > 1×10^{-6}.

Simulation under a hypothetical model of causality

To demonstrate consistent SNP association signals across mQTL, eQTL and GWAS studies under our hypothetical model of causality (**Fig. 1a**), we randomly sampled SNPs within a 1 Mb region across the genome, among which one SNP was chosen at random to be the causal variant. The methylation value (m) at the causal variant was simulated based on the model $m = zb_{zm} + c + e_{zm}$, where z is the genotype of the causal variant for an individual, c is a latent confounding variable $c \sim N(0, \sigma_c^2)$ with $\sigma_c^2 = var(zb_{zm})R_c^2/R_{zm}^2$, R_c^2 being the proportion of variance explained by c, R_{zm}^2 being the proportion of variance in m explained by z, and $e_{zm} \sim N(0, var(Zb_{zm})(1/R_{zm}^2 - 1) - \sigma_c^2)$. In our simulation, $R_c^2 = R_{zm}^2 = 0.2$, and m was standardized with mean 0 and variance 1. The expression value was simulated based on the model $x = mb_{mx} + c + e_{mx}$, where $e_{mx} \sim N(0, var(Mb_{mx})(1/R_{mx}^2 - 1) - \sigma_c^2)$ with $R_{mx}^2 = 0.2$ being the proportion of variance in x explained by m. We also standardized x with mean 0 and variance 1. We next simulated the trait phenotype based on the model $y = xb_{xy} + c + e_{xy}$, where $e_{xy} \sim N\big(0, var\big(Xb_{xy}\big)\big(1/R_{xy}^2 - 1\big) - \sigma_c^2\big)$ with $R_{xy}^2 = 0.2$ being the proportion of variance in y explained by x. We excluded the causal variant from the SNP panel and performed the mQTL, eQTL and GWAS studies. Results are shown in **Fig. 1b.**

Simulation under a linkage model

To investigate the power of HEIDI test with different number of SNPs in the test, we performed simulations under a linkage model (i.e. there are two causal variants at the locus, one for gene expression and the other for the trait) that results in heterogeneity in b_{xy} estimates. Similar to the simulation above, we randomly sampled a 1 Mb region from the genome. Among the SNPs in the region, two SNPs (z_1 and z_2) in different LD scenarios were sampled at random to be the causal variants for the gene expression and trait. The gene expression value was generated based on the model $x = z_1b_{zx} + c_x + e_{zx}$, where c_x is a latent confounding variable with $c_x \sim N\big(0, \sigma_{c_x}^2\big)$ with $\sigma_{c_x}^2 = var(z_1b_{zx})R_{cx}^2/R_{zx}^2$, $R_{cx}^2 = 0.3$ and $R_{zx}^2 = 0.3$. The trait phenotypic value was generated based on the model $y = z_2b_{zy} + c_y + e_{zy}$, where c_y is a latent confounding variable with $c_y \sim N\left(0, \sigma_{c_y}^2\right)$, $\sigma_{c_y}^2 = var\big(z_2b_{zy}\big)R_{cy}^2/R_{zy}^2$, $R_{cy}^2 = 0.3$ and $R_{zy}^2 = 0.02$. With the

simulated phenotypic data, we extracted the array SNPs (Illumina CoreExome), imputed the SNP genotypes to 1000 Genomes Project (1KGP) reference panels, and performed the SMR analysis with the 1KGP-imputed data[2]. This simulation was repeated 10,000 times and each time two causal variants were sampled with one of the nine LD scenarios (**Supplementary Fig. 13**).

Supplementary Note 2: P_{HEIDI} threshold and HEIDI power

For any statistical test that aims to remove heterogeneous observations from the data (e.g. Hardy-Weinberg Equilibrium, HWE test), it is always difficult to define an optimal threshold. For example, while a HWE test *P* value threshold of 0.01 is frequently used in GWAS, we often see studies using less conservative thresholds (e.g. 1×10^{-6}). The HEIDI threshold value ($P_{\text{HEIDI}} < 0.05$) used in Zhu *et al.*[3] was too conservative because a multiple test correction was not applied; by chance, 5% of the associations detected by the SMR test are removed even if all the associations are consistent with a pleiotropic model. Note that this proportion is expected to be higher in practice because of inevitable errors in summary-level data and heterogeneity in per-SNP sample size, especially those from multi-cohort meta-analyses. In this study, we focused on the associations that passed the SMR tests and were not rejected by the HEIDI test in all the three steps described in Method overview section (i.e. DNAm-gene, DNAm-trait and gene-trait). If we had applied a P_{HEIDI} threshold of 0.05, the HEIDI rejection rate expected under a pleiotropic model for the associations that passed the SMR test in all the three steps would be too high (approximately $1 - 0.95^3 = 14.3\%$). We therefore used a less conservative P_{HEIDI} threshold of 0.01, corresponding to an expected rejection rate of ~3% under a pleiotropic model for the results that were significant in all the three steps.

We show by simulation based on whole genome sequencing (WGS) data (**Supplementary Note 1**) that weak cis-QTL signals together with a non-stringent cis-eQTL inclusion threshold could lead to an inflated HEIDI test under a pleiotropic model owing to the inclusion of false positive cis-eQTLs (**Supplementary Fig. 12a**), resulting in decreased power in detecting pleiotropic associations. The inflation can be controlled by excluding from the HEIDI test SNPs not in LD or in low LD (e.g. $r^2 < 0.05$) with the top associated cis-eQTL (**Supplementary Fig. 12b**). Moreover, the HEIDI test involves the eigendecomposition of an $m\times m$ matrix (where m is the number of SNPs involved), which can be computationally intensive if there is a large number of SNPs included in the test. Our simulation (**Supplementary Note 1**) also shows that under a linkage model, the power of the HEIDI test initially increases with m (the top m associated SNPs after pruning for strong LD[3]), reaches the maximum when $m\approx26$ for WGS SNPs and $m\approx14$ for HapMap2 SNPs (**Supplementary Fig. 13**). We therefore recommend the use of m=20 in practice to improve the computational efficiency of large-scale analysis (e.g., the analysis of mapping the

methylome to the transcriptome below) without sacrificing the power of heterogeneity detection. In addition, it has been shown previously[3] and confirmed by our simulation (**Supplementary Fig. 13**) that the HEIDI test cannot reject a linkage model if the two causal variants are in perfect LD, and the rejection power decreases with the increase of LD between the two causal variants. It should also be noted that we do not attempt to test for causality because SMR analysis using SNP instrument at a single locus is unable to distinguish pleiotropy from causality[3].

Supplementary Note 3: Test for the difference in variance explained

For the top associated cis-eQTL, we can estimate the SNP effect and standard error with respect to the standardised expression level (or DNAm level) based on z score and allele frequency (p) using the method described in Zhu et al.[3], i.e.

$\hat{b} = z/\sqrt{2p(1-p)(n+z^2)}$ and $se = 1/\sqrt{2p(1-p)(n+z^2)}$.

The variance explained by the top eQTL can be estimated as $\hat{q}^2 = 2p(1-p)\hat{b}^2$. We define the difference of $\hat{q}^2$ as $\hat{d}_i = \hat{q}^2_{1(i)} - \hat{q}^2_{2(i)}$, where $\hat{q}^2_1$ and $\hat{q}^2_2$ are the estimated variance in gene expression and DNAm explained by the top eQTL respectively. The variance of $\hat{d}_i$ can be calculated following $var(\hat{d}_i) = var(\hat{q}^2_{1(i)}) + var(\hat{q}^2_{2(i)})$ assuming the eQTL and mQTL are estimated from independent studies, where the sampling variance of $\hat{q}^2$ can be approximately calculated as $var(\hat{q}^2) = 16p^2(1-p)^2\,\hat{b}^2 se^2$ using the Delta method. The significance of $\hat{d}_i$ can therefore be assessed by a Wald test, i.e., $\frac{\hat{d}_i^2}{var(\hat{d}_i)} \sim \chi^2_1$.

Supplementary Note 4: Benefits of incorporating epigenetic data

There are several advantages of integrating epigenetic data into the SMR & HEIDI analysis. First, the pleiotropic associations of DNAm with both gene expression and trait reinforce our confidence in the functional relevance of the genes identified by SMR & HEID with the trait. Second, the integration of DNAm and epigenomic annotation data helps us to better understand and hypothesize the regulatory mechanisms at a GWAS locus (**Fig. 4** and **5**; **Supplementary Fig. 8**). Third, the integrative analysis is also helpful to narrow down the genomic regions where the causal variants are possibly located (see the *FADS2* example in **Fig. 4** where rs968567 is likely to be a causal variant regulating the expression level of one of the *FADS2* isoforms and the *ATG16L1* example in **Fig. 5** where rs2241880 has confirmed to be a causal variant for *Atg16l1* in mice[4]). However, the benefit of adding DNAm data is at the cost of statistical power if we only focus on the associations passing the SMR test and not rejected by the HEIDI test in all the three steps illustrated in **Fig. 1**.

Supplementary Note 5: Acknowledgments

HRS (dbGaP accession: phs000428.v1.p1): HRS is supported by the National Institute on Aging (NIA U01AG009740). The genotyping was funded separately by the National Institute on Aging (RC2 AG036495, RC4 AG039029). Genotyping was conducted by the NIH Center for Inherited Disease Research (CIDR) at Johns Hopkins University. Genotyping quality control and final preparation of the data were performed by the Genetics Coordinating Center at the University of Washington.

ARIC (dbGaP accession: phs000280.v4.p1): The Atherosclerosis Risk in Communities Study is carried out as a collaborative study supported by National Heart, Lung, and Blood Institute contracts (HHSN268201100005C, HHSN268201100006C, HHSN268201100007C, HHSN268201100008C, HHSN268201100009C, HHSN268201100010C, HHSN268201100011C, and HHSN268201100012C), R01HL087641, R01HL59367 and R01HL086694; National Human Genome Research Institute contract U01HG004402; and National Institutes of Health contract HHSN268200625226C. The authors thank the staff and participants of the ARIC study for their important contributions. Infrastructure was partly supported by Grant Number UL1RR025005, a component of the National Institutes of Health and NIH Roadmap for Medical Research.

UK10K (EGA accessions: EGAS00001000108 and EGAS00001000090): The UK10K project was funded by the Wellcome Trust award WT091310. Twins UK (TUK): TUK was funded by the Wellcome Trust and ENGAGE project grant agreement HEALTH-F4-2007–201413. The study also receives support from the Department of Health via the National Institute for Health Research (NIHR)-funded BioResource, Clinical Research Facility and Biomedical Research Centre based at Guy's and St. Thomas' NHS Foundation Trust in partnership with King's College London. Dr Spector is an NIHR senior Investigator and ERC Senior Researcher. Funding for the project was also provided by the British Heart Foundation grant PG/12/38/29615 (Dr Jamshidi). A full list of the investigators who contributed to the UK10K sequencing is available from http://www.UK10K.org.

CMC (Synapse accession: syn2759792): CMC data were generated as part of the CommonMind Consortium supported by funding from Takeda Pharmaceuticals Company Limited, F. Hoffman-La Roche Ltd and NIH grants R01MH085542, R01MH093725, P50MH066392, P50MH080405, R01MH097276, RO1-MH-075916, P50M096891, P50MH084053S1, R37MH057881 and R37MH057881S1, HHSN271201300031C, AG02219, AG05138 and MH06692. Brain tissue for the study was obtained from the following brain bank collections: the Mount Sinai NIH Brain and Tissue Repository, the University of Pennsylvania Alzheimer's Disease Core Center, the

University of Pittsburgh NeuroBioBank and Brain and Tissue Repositories and the NIMH Human Brain Collection Core. CMC Leadership: Pamela Sklar, Joseph Buxbaum (Icahn School of Medicine at Mount Sinai), Bernie Devlin, David Lewis (University of Pittsburgh), Raquel Gur, Chang-Gyu Hahn (University of Pennsylvania), Keisuke Hirai, Hiroyoshi Toyoshiba (Takeda Pharmaceuticals Company Limited), Enrico Domenici, Laurent Essioux (F. Hoffman-La Roche Ltd), Lara Mangravite, Mette Peters (Sage Bionetworks), Thomas Lehner, Barbara Lipska (NIMH).

GTEx (dbGaP accession: phs000424.v6.p1): The Genotype-Tissue Expression (GTEx) Project was supported by the Common Fund of the Office of the Director of the National Institutes of Health (commonfund.nih.gov/GTEx). Additional funds were provided by the NCI, NHGRI, NHLBI, NIDA, NIMH, and NINDS. Donors were enrolled at Biospecimen Source Sites funded by NCI Leidos Biomedical Research, Inc. subcontracts to the National Disease Research Interchange (10XS170), Roswell Park Cancer Institute (10XS171), and Science Care, Inc. (X10S172). The Laboratory, Data Analysis, and Coordinating Center (LDACC) was funded through a contract (HHSN268201000029C) to the The Broad Institute, Inc. Biorepository operations were funded through a Leidos Biomedical Research, Inc. subcontract to Van Andel Research Institute (10ST1035). Additional data repository and project management were provided by Leidos Biomedical Research, Inc.(HHSN261200800001E). The Brain Bank was supported supplements to University of Miami grant DA006227. Statistical Methods development grants were made to the University of Geneva (MH090941 & MH101814), the University of Chicago (MH090951, MH090937, MH101825, & MH101820), the University of North Carolina - Chapel Hill (MH090936), North Carolina State University (MH101819), Harvard University (MH090948), Stanford University (MH101782), Washington University (MH101810), and to the University of Pennsylvania (MH101822)

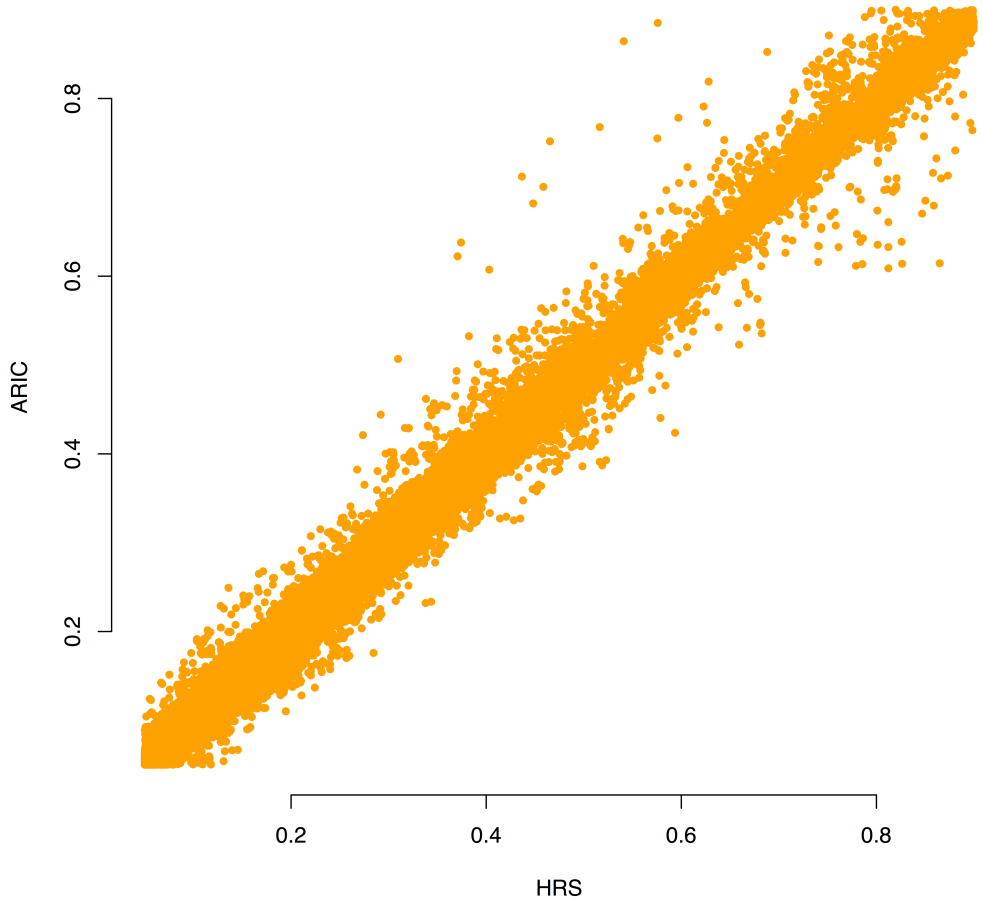

Supplementary Figure 1 Consistency of LD r^2 between two samples of European ancestry. Shown are the LD r^2 between 1500 pairs of adjacent common SNPs on chromosome 22 estimated in the HRS[5] (n = 8,479 unrelated European Americans) and ARIC[6] data (n = 7,703 unrelated European Americans). The comparison was limited to SNP pairs with $0.05 < r^2 < 0.9$ in both data sets.

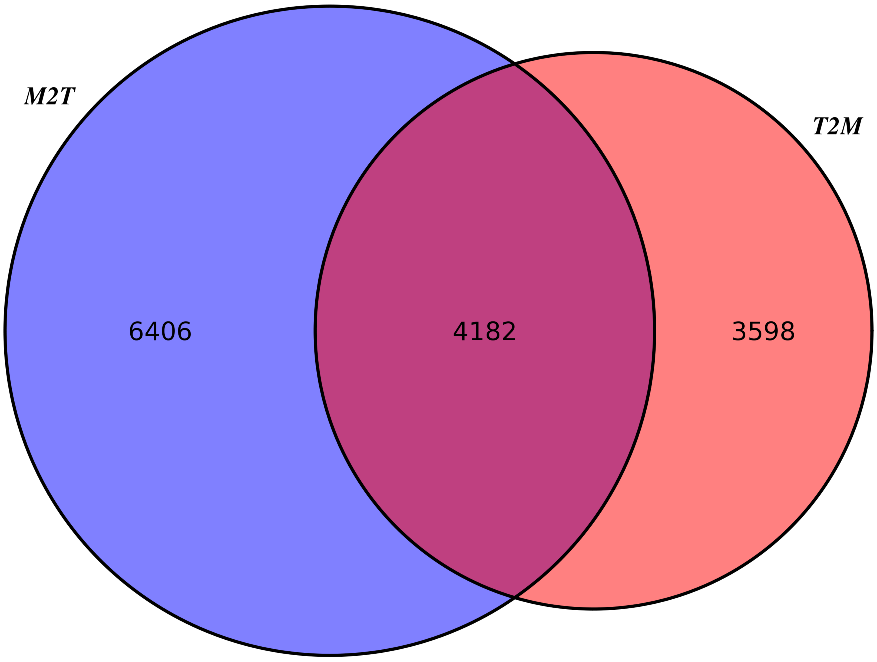

Supplementary Figure 2 DNAm-gene associations identified by the SMR and HEIDI analysis with different strategies. M2T: SMR and HEIDI analysis considering DNAm as the exposure and gene expression as the outcome. T2M: SMR and HEIDI analysis considering gene expression as the exposure and DNAm as the outcome.

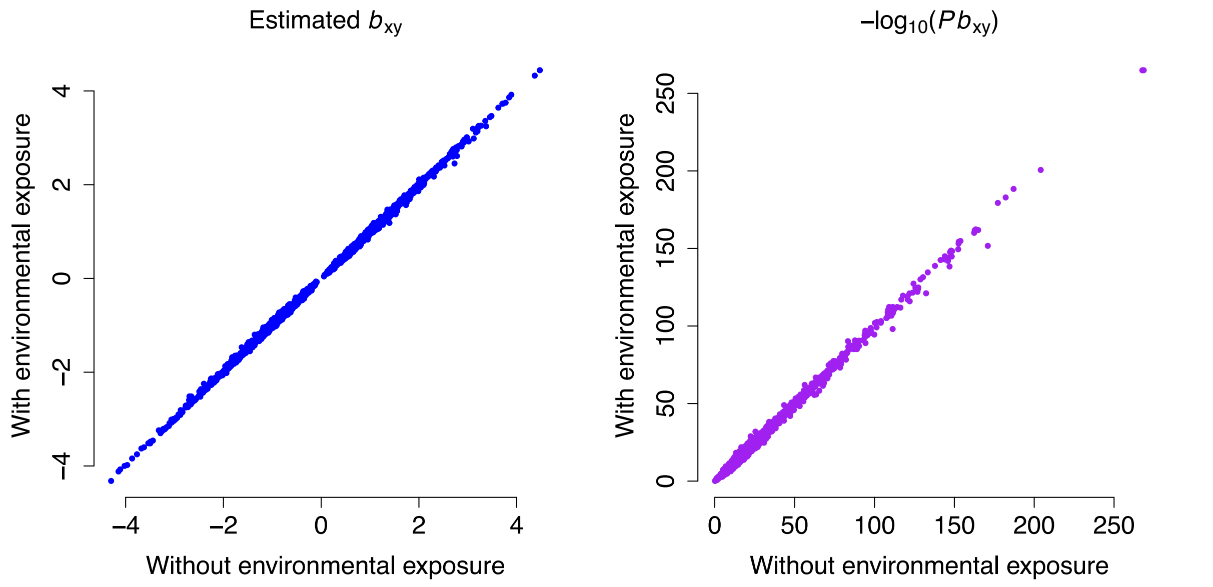

Supplementary Figure 3 SMR estimates of the effect of DNAm on expression with and without environmental exposure. We simulated data using a method similar to that described in **Supplementary Note 1**. We randomly sampled a SNP from the UK10K-WGS data as the causal variant and generated the DNAm (x) and gene expression (y) data based on the model $x = zb_{zx} + e_{zx}$ and $y = zb_{zy} + e_{zy}$ where the SNP effects (b_{zx} and b_{zy}) and the proportion of variance in x and y explained by the SNP (R^2_{zx} and R^2_{zy}) were randomly sampled from the estimates in the CAGE eQTL summary data[7] and the McRae et al. mQTL summary data[8], respectively. We then simulated an environmental exposure that was also affected by the SNP based on the model $l = zb_{zl} + e_{zl}$ with $R^2_{zl} = 0.01$. The effect of the environmental exposure on gene expression was then modeled as $y^* = \mathrm{y} + lb_{ly} + c$, where c is the mean of y and $b_{ly} = \sqrt{\mathrm{var}(y)/\mathrm{var}(l)}$, mimicking a scenario that both mean and variance of y were doubled. We repeated the simulation 5,000 times and compared the SMR estimates and the corresponding SMR P values between the two scenarios (i.e. with or without the environmental exposure).

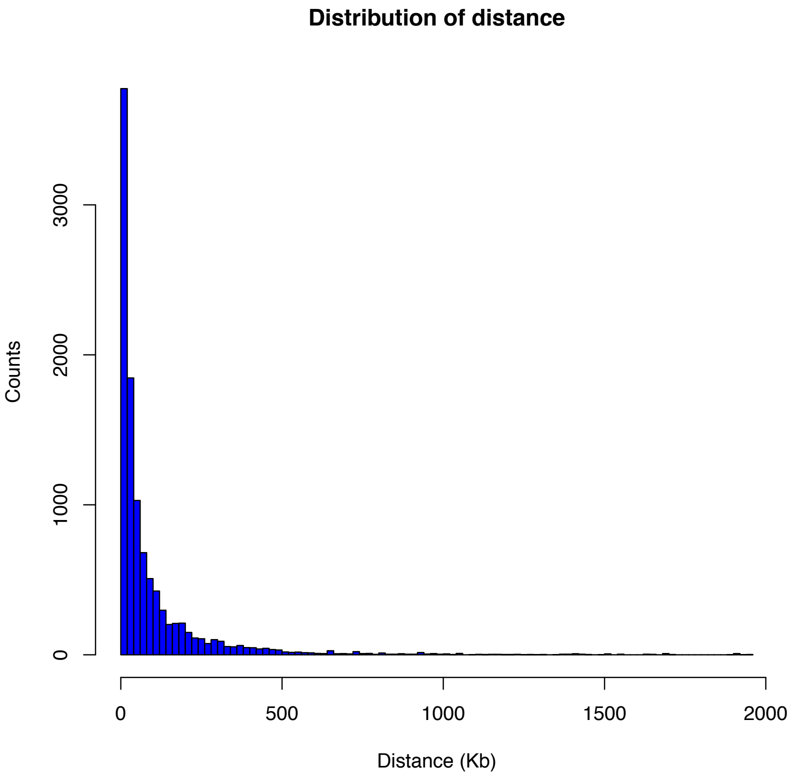

Supplementary Figure 4 Distribution of physical distance between the DNAm sites and the associated transcripts identified in the SMR and HEIDI tests (10,588 pairs in total).

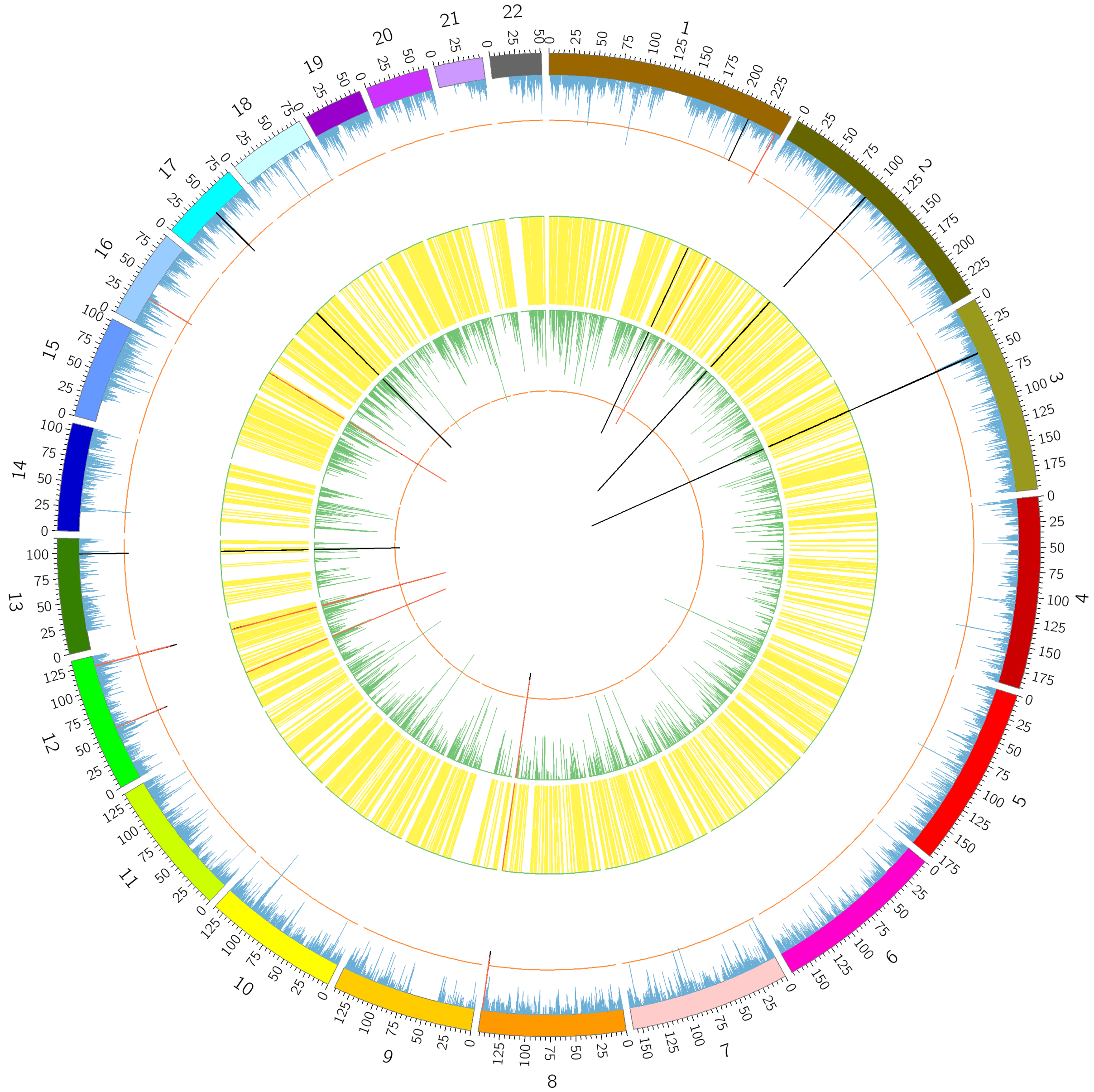

Supplementary Figure 5 Pinpointing functional regions for a complex trait with consistent SMR association signals across multi-omics layers. Shown are -$\log_{10}$(P values) values for educational years (EY) against the physical positions of DNAm or gene expression probes. The blue lines (outer ring) represent -$\log_{10}$(P values) from SMR tests for associations between DNA methylation and trait, the green lines (inner ring) represent those for associations between transcripts and trait, and the yellow lines (middle ring) represent the significant associations between methylations and transcripts. The orange circles represent the significance thresholds of the SMR test. The black lines are the significant SMR associations consistent across all three layers, and red lines highlight the significant and consistent SMR associations that are not rejected by the HEIDI test.

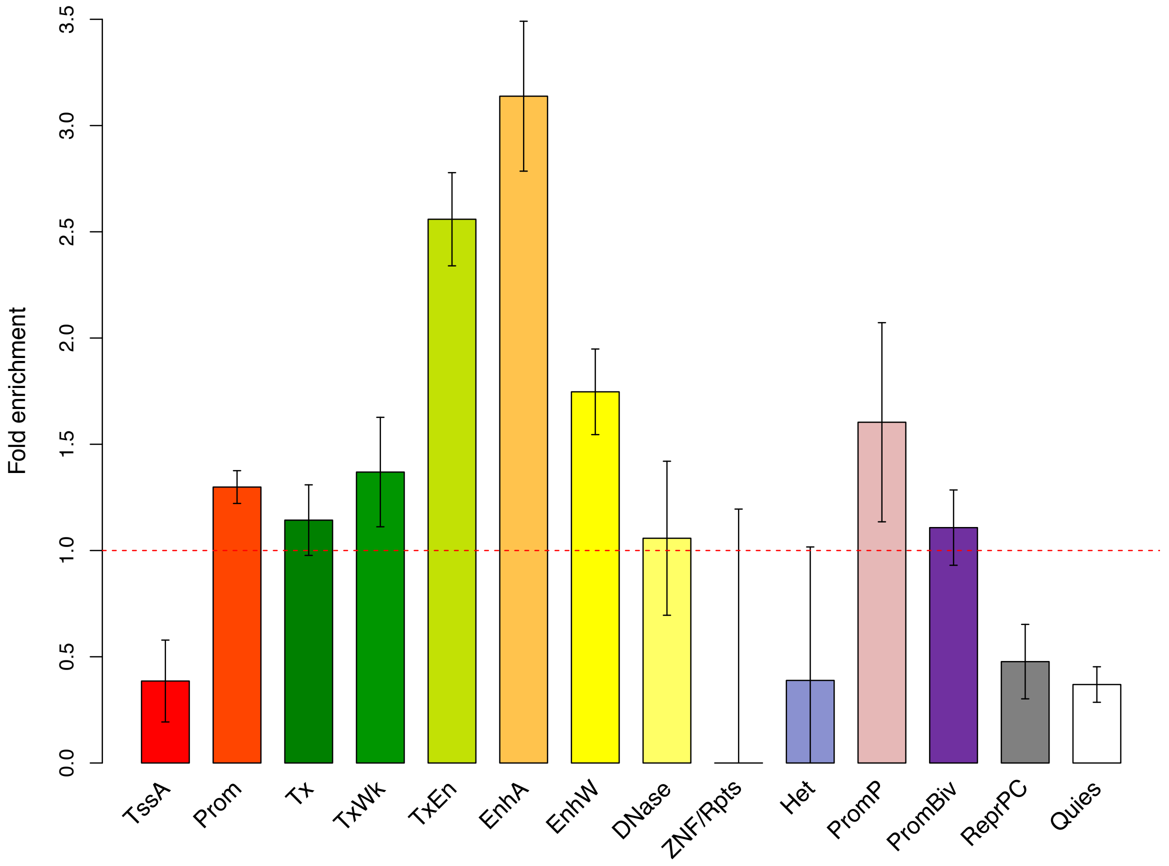

Supplementary Figure 6 Enrichment analysis of 149 DNAm probes significantly associated with both expression and traits identified by the SMR/HEIDI test for 14 main functional annotation categories. Shown are the comparison of the associated probes with the same probes sampled repeatedly at random with the variance of each probe matched. Error bar represents the standard error of an estimate obtained from 500 random samples. The 14 functional categories are: TssA, active transcription start site; Prom, upstream/downsteam TSS promoter; Tx, actively transcribed state; TxWk, weak transcription; TxEn, transcribed and regulatory Prom/Enh; EnhA, active enhancer; EnhW, weak enhancer; DNase, primary DNase; ZNF/Rpts, state associated with zinc finger protein genes; Het, constitutive heterochromatin; PromP, Poised promoter; PromBiv, bivalent regulatory states; ReprPC, repressed Polycomb states; and Quies, a quiescent state.

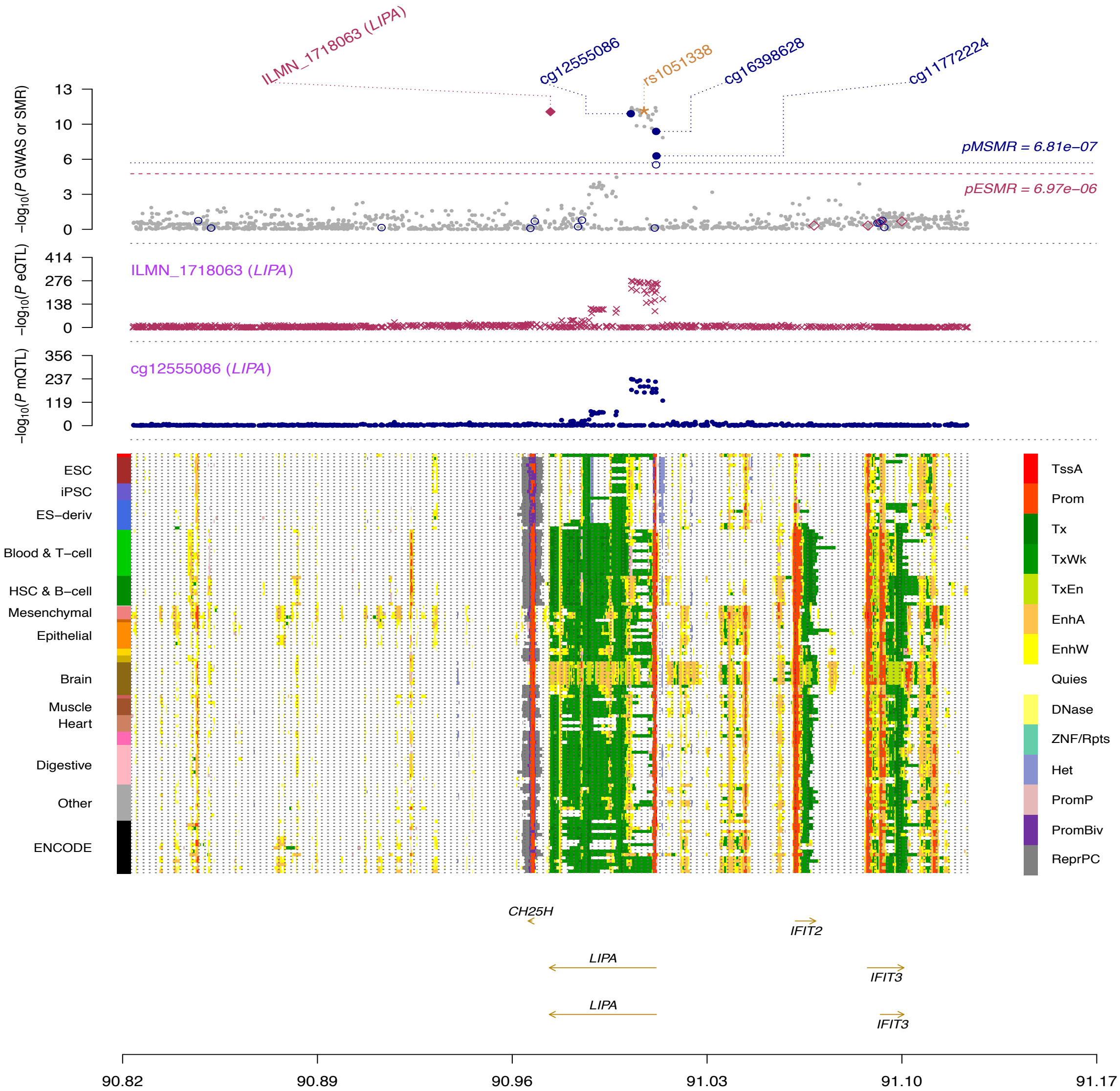

Supplementary Figure 7 Prioritizing genes and regulatory elements for coronary artery disease (CAD) at a GWAS locus with an inferred regulation mechanism. The top plot shows the -log$_{10}$(P values) of SNPs from the latest GWAS meta-analysis for CAD. The red diamonds and blue circles represent the -log$_{10}$(P values) from SMR tests for associations of gene expression and DNAm probes with CAD, respectively. The solid circles are the probes not rejected by the HEIDI test. The yellow star shows the previously reported causal variant rs1051338 (denoted in yellow/gold). The second plot shows -log$_{10}$(P values) of the SNP-association for gene expression probe ILMN_1718063 (tagging *LIPA*) from the CAGE eQTL study. The third plot shows -log$_{10}$(P values) of the SNP-association for DNAm probe cg12555086 from the mQTL study. The bottom plot shows 14 chromatin state annotations (indicated by colours) of 127 samples from REMC for different primary cells and tissue types (rows).

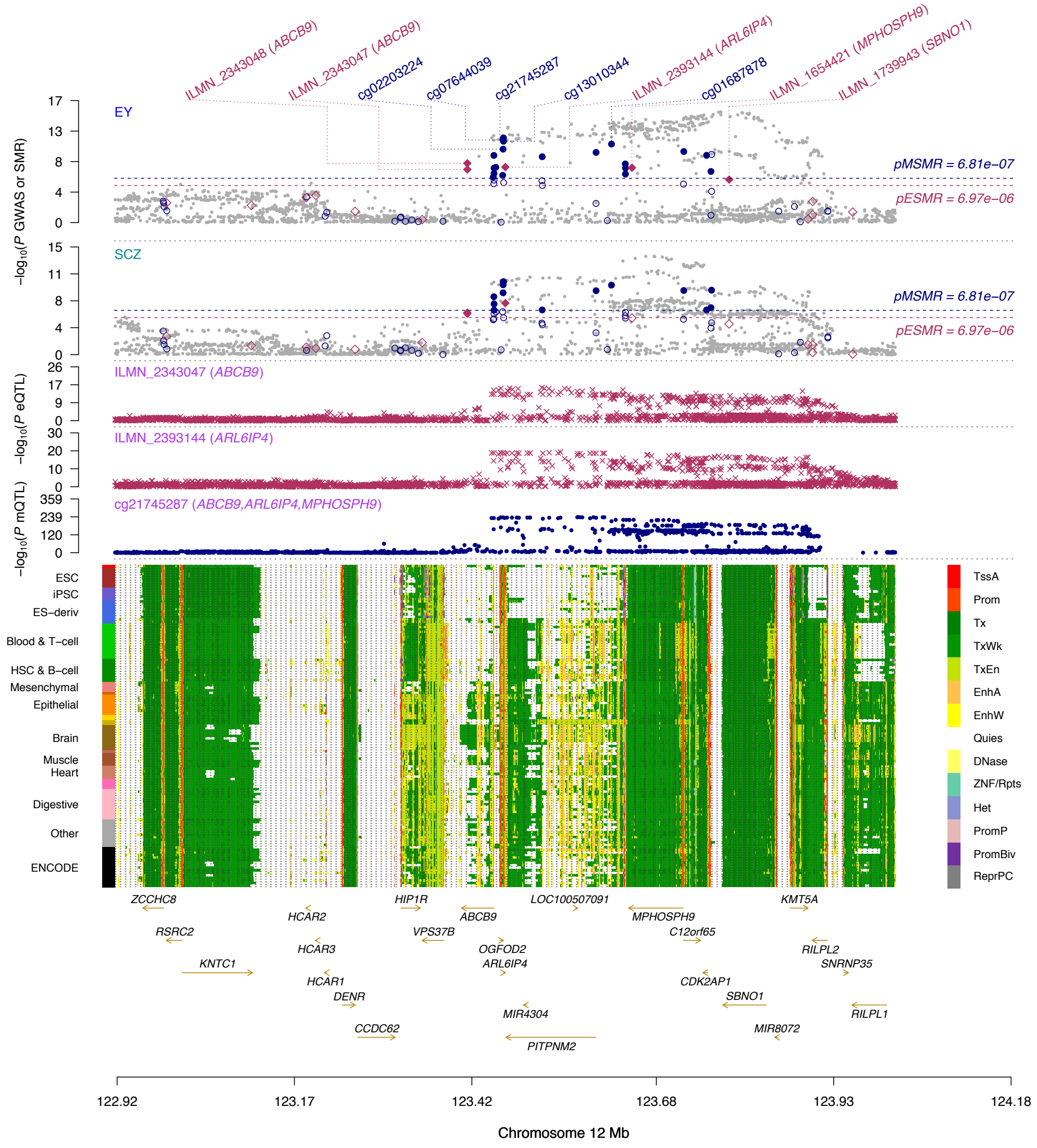

Supplementary Figure 8 Pleiotropic genes and DNAm sites shared by multiple traits. Shown are the results at *ARL6IP4* locus for educational years (EY) and schizophrenia (SCZ). The top plot shows the -log$_{10}$(P values) of SNPs from GWAS summary data for EY and SCZ. The red diamonds and blue circles represent the -log$_{10}$(P values) from SMR tests for associations of gene expression and DNAm probes with EY and SCZ, respectively. The solid circles are the probes not rejected by the HEIDI test. The second plot shows -log$_{10}$(P values) of the SNP-association for gene expression probes ILMN_2343047 (tagging *ABCB9*) and ILMN_2393144 (tagging *ARL6IP4*) from the CAGE eQTL study. The third plot shows -log$_{10}$(P values) of the SNP-association for DNAm probe cg021745287 from the mQTL meta-analysis. The bottom plot shows 14 chromatin state annotations (indicated by colours) of 127 samples from REMC for different primary cells and tissue types (rows).

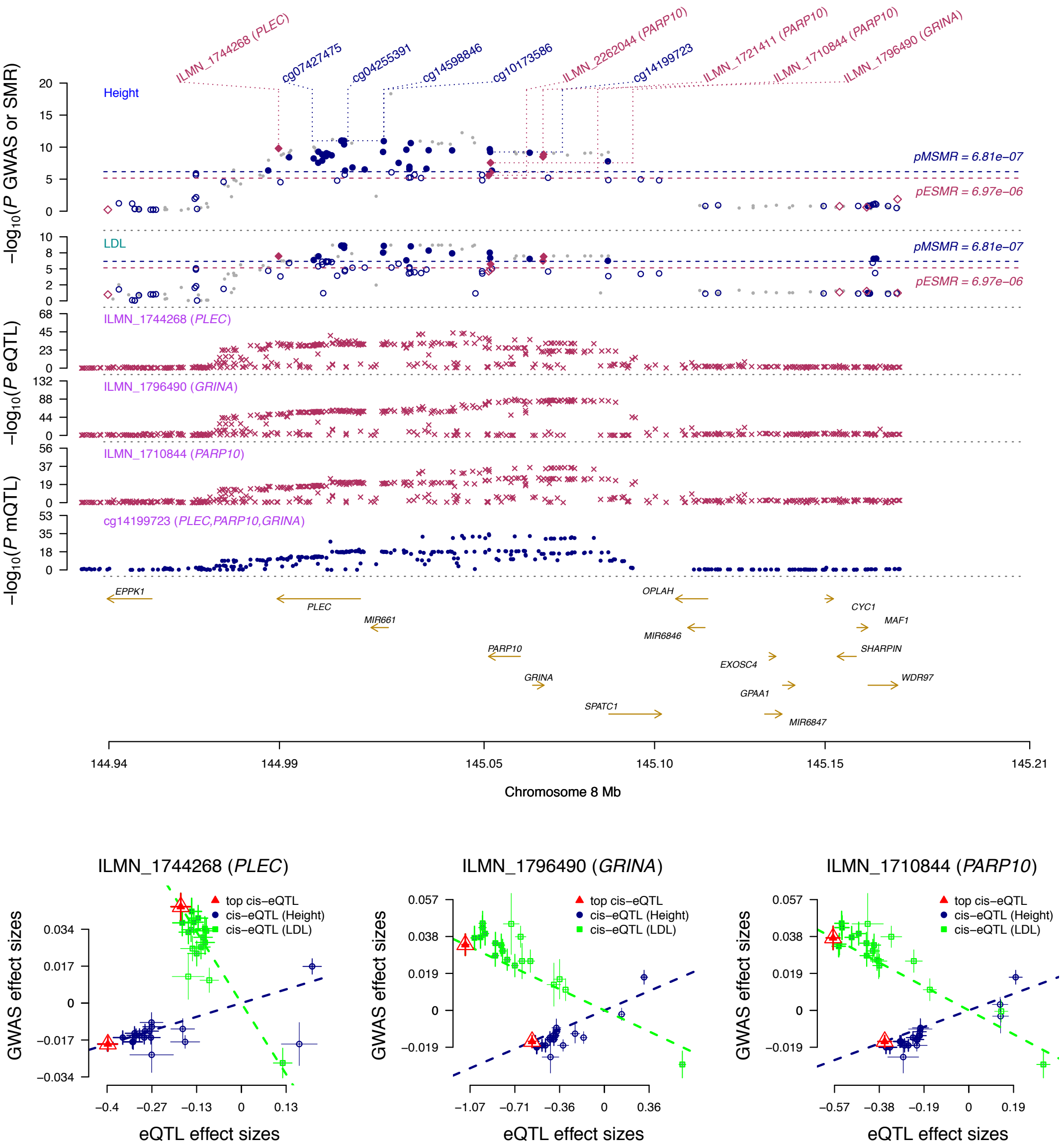

Supplementary Figure 9 Genes and DNAm showing pleiotropic effects in opposite directions on height and low-density lipoprotein (LDL). Shown are the results at the *PLEC*, *GRINA* and *PARP10* loci for height and LDL. The grey points are the SNP-association -$\log_{10}$(P values) from GWAS summary data for height and LDL. Red diamonds and blue circles in the top layer (two plots) represent the -$\log_{10}$(P values) from the SMR tests for associations of gene expression and DNAm probes with the trait, respectively. Solid circles are those not rejected by the HEIDI test. Shown in the second layer (three plots; the red crosses) are -$\log_{10}$(P values) of the SNP-association from the CAGE eQTLs study for probes ILMN_1744268, ILMN_1710844 and ILMN_1796490. The blue dots at the third layer are -$\log_{10}$(P values) of the mQTL for the three associated DNAm probes. The bottom plots show the estimates of eQTL effects against their estimated effects on height and LDL. The dashed lines represent the estimate of b_{SMR} at the top *cis*-eQTL (rather than the regression line).

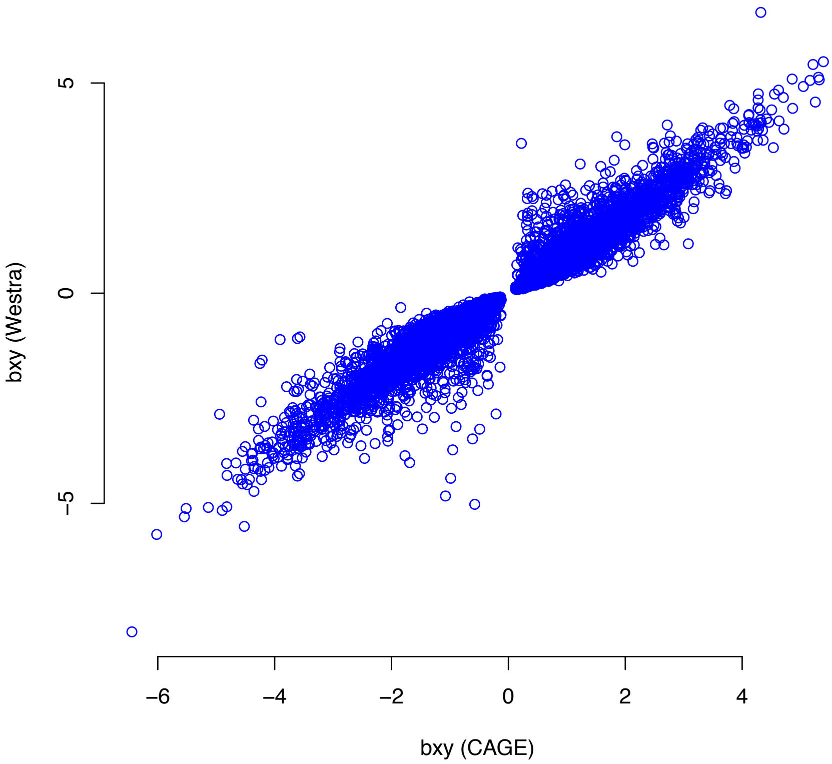

Supplementary Figure 10 Consistent SMR effect estimates of DNAm (x) on transcripts (y) when eQTL summary data are from different studies (CAGE versus Westra).

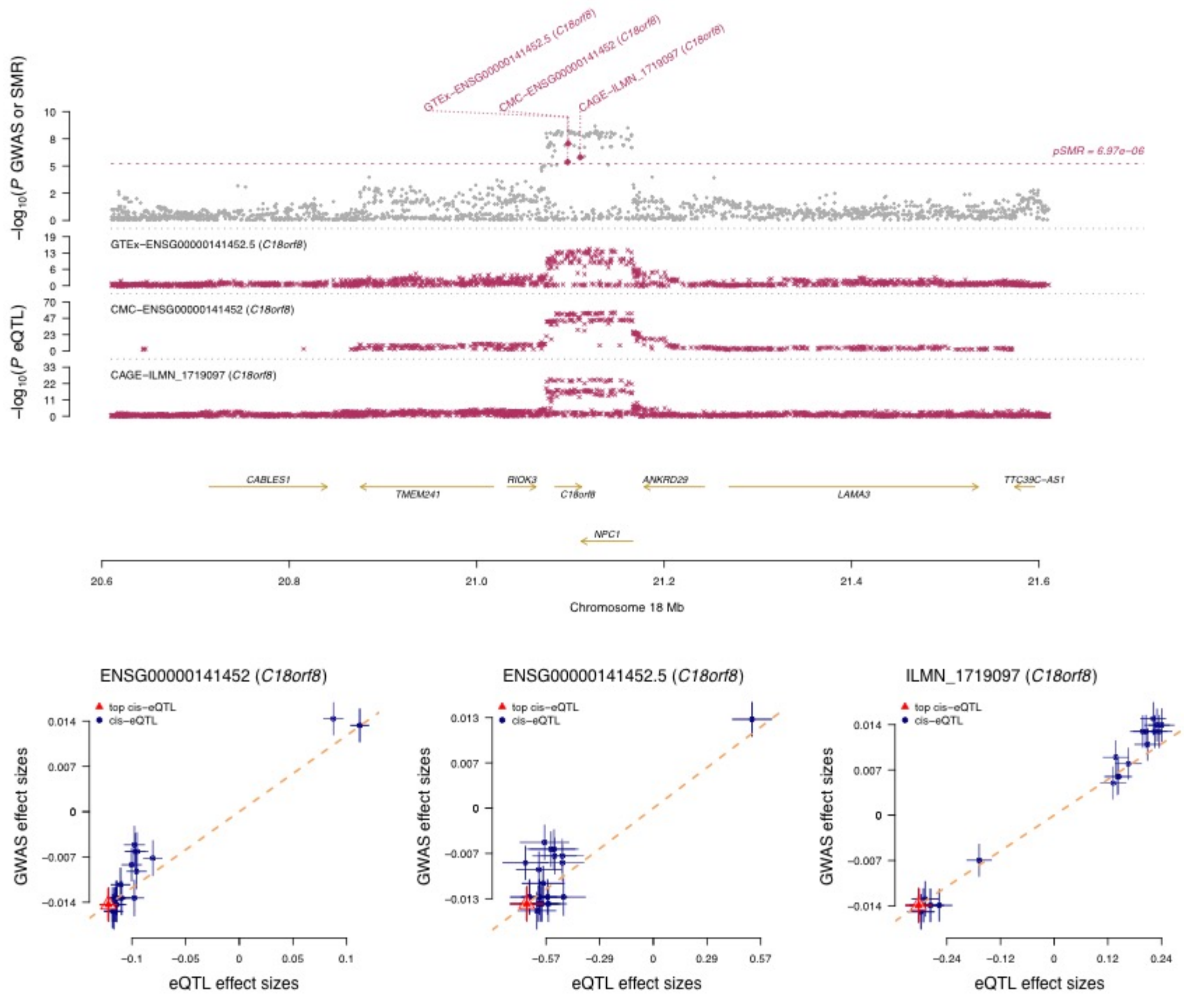

Supplementary Figure 11 Replication analysis in the *C18orf8* locus for educational years (EY). A) The top plot shows the SNP-association -log$_{10}$(*P* values) from the latest GWAS meta-analysis for EY. Red diamonds represent the -log$_{10}$(*P* values) from the SMR tests for associations of gene expression with EY. Solid ones are the probes not rejected by the HEIDI test. Shown in the second layer (three plots; the red crosses) are the -log$_{10}$(*P* values) SNP-association from three datasets (GTEx, CMC, CAGE). The bottom plots show the estimates of eQTL effects against their estimated effects on EY. The orange dashed lines represent the estimate of b_{SMR} at the top *cis*-eQTL (rather than the regression line).

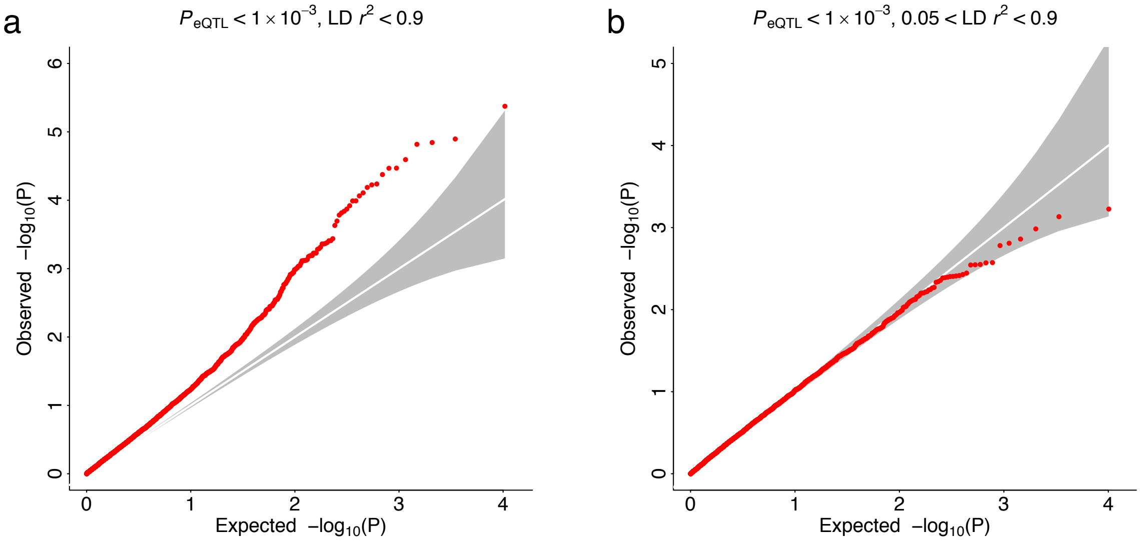

Supplementary Figure 12 QQ plots of the HEIDI test *P* values under a pleiotropy model. Shown are the results from simulations based on the UK10K-WGS data (**Supplementary Note 1**). In brief, we randomly sampled a sequence variant as the causal variant and simulated the expression value based on the model $x = zb_{zx} + e_{zx}$ with R^2_{zx} (variance explained by the causal variant) estimated from the CAGE eQTL summary data. The trait value was generated based on the model $y = zb_{zy} + e_{zy}$ with $R^2_{zy} = 0.01$. We then performed the SMR test and carried the associations with $P_{SMR} < 1\times10^{-6}$ forward for the HEIDI test. The HEIDI test was performed with different criteria to filter SNPs. In panel a, we selected SNPs with $P_{eQTL} < 1\times10^{-3}$, removed SNPs in LD $r^2 > 0.9$ with the top cis-eQTL, and included the top 20 SNPs (ranked by P_{eQTL}) in the HEIDI test. In panel b, we added an additional filtering step, i.e. removing SNPs in LD $r^2 < 0.05$ with the top cis-eQTL. We repeated the simulation 10,000 times.

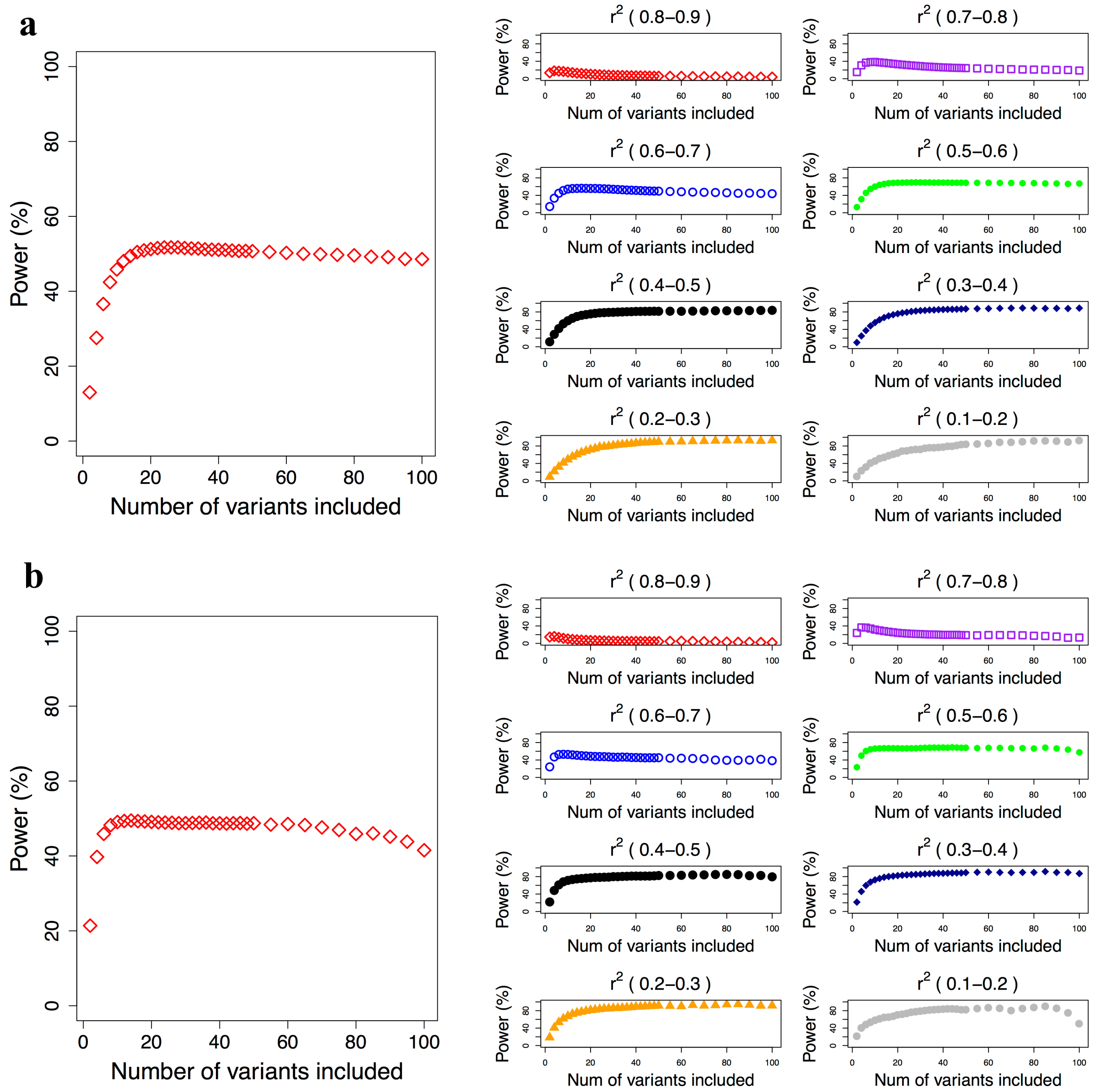

Supplementary Figure 13 Power of the HEIDI test to detect heterogeneity changes with the number of SNPs included in the test. The *x*-axis represents the number of SNPs included in the HEIDI test and *y*-axis is the power of detecting heterogeneity at HEIDI *P* value < 0.01. The results are from simulations based on UK10K-WGS data (**Supplementary Note 1**). The summary-level statistics and LD were obtained from 1000G-imputed data in panel a and HapMap2-imputed data in pane b. Only the associations with SMR *P* value < 1×10^{-6} were carried forward for the HEIDI test. The associations with *HEIDI P* value < 0.01 were considered as those with significant detection of heterogeneity. In each panel, shown in the plots on the right were the results stratified by LD r^2 between the two causal variants at the locus, and shown in the plot on the left were the results of the whole set.

Supplementary Table 1 Definition of 25/14 functional categories from 127 Epigenome reference samples.

Chromatin State (25)	Chromatin State Annotation (25)	Chromatin State (14)	Chromatin State Annotation (14)
1 TssA	Active TSS	1 TssA	Active TSS
2 PromU	Promoter Upstream TSS	2 Prom	Promoter Upstream/Downstream TSS
3 PromD1	Promoter Downstream TSS 1		
4 PromD2	Promoter Downstream TSS 2		
5 Tx5'	Transcribed and 5' preferential	3 Tx	Active transcription
6 Tx	Strong transcription		
7 Tx3'	Transcribed and 3' preferential		
8 TxWk	8 Weak transcription	4 TxWk	Weak transcription
9 TxReg	Transcribed&regulatory (Prom/Enh)	5 TxEn	Transcribed and regulatory Prom/Enh
10 TxEnh5'	Transcribed 5' preferential and Enh		
11 TxEnh3'	Transcribed 3' preferential and Enh		
12 TxEnhW	Transcribed and weak Enhancer		
13 EnhA1	Active Enhancer 1	6 EnhA	Active enhancer
14 EnhA2	Active Enhancer 2		
15 EnhAF	Active Enhancer Flank		
16 EnhW1	Weak Enhancer 1	7 EnhW	Weak enhancer
17 EnhW2	Weak Enhancer 2		
18 EnhAc	Primary H3K27ac possible Enhancer		
19 DNase	Primary DNase	8 DNase	Primary DNase
20 ZNF/Rpts	ZNF genes & repeats	9 ZNF/Rpts	ZNF genes & repeats
21 Het	Heterochromatin	10 Het	Heterochromatin
22 PromP	Poised Promoter	11 PromP	Poised Promoter
23 PromBiv	BivalentPromoter	12 PromBiv	BivalentPromoter
24 ReprPC	Repressed PolyComb	13 ReprPC	Repressed PolyComb
25 Quies	Quiescent/Low	14 Quies	Quiescent/Low

Supplementary Reference

1. UK10K Consortium *et al.* The UK10K project identifies rare variants in health and disease. *Nature* **526**, 82-90 (2015).
2. Yang, J. *et al.* Genetic variance estimation with imputed variants finds negligible missing heritability for human height and body mass index. *Nat Genet* **47**, 1114-1120 (2015).
3. Zhu, Z. *et al.* Integration of summary data from GWAS and eQTL studies predicts complex trait gene targets. *Nat Genet* **48**, 481-7 (2016).
4. Murthy, A. *et al.* A Crohn's disease variant in Atg16l1 enhances its degradation by caspase 3. *Nature* **506**, 456-62 (2014).
5. Sonnega, A. *et al.* Cohort Profile: the Health and Retirement Study (HRS). *Int J Epidemiol* **43**, 576-85 (2014).
6. The Atherosclerosis Risk in Communities (ARIC) Study: design and objectives. The ARIC investigators. *Am J Epidemiol* **129**, 687-702 (1989).
7. Lloyd-Jones, L.R. *et al.* The Genetic Architecture of Gene Expression in Peripheral Blood. *Am J Hum Genet* **100**, 371 (2017).
8. McRae, A. *et al.* Identification of 55,000 Replicated DNA Methylation QTL. *bioRxiv* (2017).
