# Supplementary material for: Integrative analysis of omics summary data reveals putative mechanisms underlying complex traits
Source: Nat Commun. 2018 Mar 2;9:918. doi: 10.1038/s41467-018-03371-0 (PMC5834629; doi:10.1038/s41467-018-03371-0)
Supplement: Supplementary file 2 — Description of Additional Supplementary Files [file 41467_2018_3371_MOESM2_ESM.docx]

**Description of Additional Supplementary Files**

**File Name: Supplementary Data 1**

Description: DNAm-gene associations for which the top cis-eQTL explains significantly more proportion of variance in gene expression than in DNAm.

**File Name: Supplementary Data 2**

Description: Descriptive summary of the GWAS data sets used in this analysis.

**File Name: Supplementary Data 3**

Description: Identification of 374 genes by the SMR analysis for 13 complex traits.

**File Name: Supplementary Data 4**

Description: Identification of 564 genes by multi-SNP based SMR analysis for 13 complex traits.

**File Name: Supplementary Data 5**

Description: Identification of 1,903 DNAm sites by the SMR analysis for 14 complex traits.

**File Name: Supplementary Data 6**

Description: Pleiotropic associations between DNAm sites, transcripts and complex traits from the SMR analyses of multiple omics data.

**File Name: Supplementary Data 7**

Description: Prioritized associations between 149 DNAm sites, 66 genes and 12 complex traits.

**File Name: Supplementary Data 8**

Description: Identification of 5 genes overlapped with drug targets.

**File Name: Supplementary Data 9**

Description: Pleiotropic effects of DNAm sites and transcripts on multiple traits.

**File Name: Supplementary Data 10**

Description: Replications of the SMR results for schizophrenia and educational years in different tissues and data sets.
